# Engineered Cell Elongation Promotes Extracellular Electron Transfer of Shewanella Oneidensis

**DOI:** 10.1002/advs.202403067

**Published:** 2024-09-05

**Authors:** Feng Li, Huan Yu, Baocai Zhang, Chaoning Hu, Fei Lan, Yuxuan Wang, Zixuan You, Qijing Liu, Rui Tang, Junqi Zhang, Chao Li, Liang Shi, Wen‐Wei Li, Kenneth H. Nealson, ZhanYing Liu, Hao Song

**Affiliations:** ^1^ Frontier Science Center for Synthetic Biology (Ministry of Education) Key Laboratory of Systems Bioengineering and School of Chemical Engineering and Technology Tianjin University Tianjin 300072 China; ^2^ Department of Biological Sciences and Technology School of Environmental Studies China University of Geoscience in Wuhan Wuhan Hubei 430074 China; ^3^ Chinese Academy of Sciences Key Laboratory of Urban Pollutant Conversion Department of Environmental Science and Engineering University of Science & Technology of China Hefei 230026 China; ^4^ Departments of Earth Science & Biological Sciences University of Southern California 4953 Harriman Ave. South Pasadena CA 91030 USA; ^5^ Center for Energy Conservation and Emission Reduction in Fermentation Industry in Inner Mongolia Engineering Research Center of Inner Mongolia for Green Manufacturing in Bio‐fermentation Industry and School of Chemical Engineering Inner Mongolia University of Technology Inner Mongolia Hohhot 010051 China; ^6^ Haihe Laboratory of Sustainable Chemical Transformations Tianjin 300192 China

**Keywords:** biofilm formation, cellular length, *c*‐type cytochromes, divisome, extracellular electron transfer (EET)

## Abstract

To investigate how cell elongation impacts extracellular electron transfer (EET) of electroactive microorganisms (EAMs), the division of model EAM *Shewanella oneidensis* (*S. oneidensis*) MR‐1 is engineered by reducing the formation of cell divisome. Specially, by blocking the translation of division proteins via anti‐sense RNAs or expressing division inhibitors, the cellular length and output power density are all increased. Electrophysiological and transcriptomic results synergistically reveal that the programmed cell elongation reinforces EET by enhancing NADH oxidation, inner‐membrane quinone pool, and abundance of *c*‐type cytochromes. Moreover, cell elongation enhances hydrophobicity due to decreased cell‐surface polysaccharide, thus facilitates the initial surface adhesion stage during biofilm formation. The output current and power density all increase in positive correction with cellular length. However, inhibition of cell division reduces cell growth, which is then restored by quorum sensing‐based dynamic regulation of cell growth and elongation phases. The QS‐regulated elongated strain thus enables a cell length of 143.6 ± 40.3 µm (72.6‐fold of that of *S. oneidensis* MR‐1), which results in an output power density of 248.0 ± 10.6 mW m^−2^ (3.41‐fold of that of *S. oneidensis* MR‐1) and exhibits superior potential for pollutant treatment. Engineering cellular length paves an innovate avenue for enhancing the EET of EAMs.

## Graphical abstract

This work investigates how cell elongation impacts the extracellular electron transfer of *Shewanella oneidensis* MR‐1 via genetic engineering to inhibit divisome formation. The electrophysiology of cell elongation is systematically elucidated in terms of intracellular generation, trans‐membrane transfer, and biofilm formation. Moreover, quorum sensing‐based dynamic regulation is integrated to separate cell growth and elongation phases and regain cell growth.

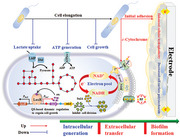

## 1 Introduction

EAMs exchange electrons and energy with environments via EET, which play a central role in diverse bio‐electrochemical systems (BES) for sustainable power and chemicals production.^[^ ^1^ ^]^ For example, microbial fuel cells (MFC) utilize electron‐producing bacteria to produce bioelectricity via degradation of organic wastes in wastewaters,^[^ ^2^ ^]^ while electron‐harvesting bacteria are used for CO_2_ and N_2_ fixation,^[^ ^3^ ^]^ to achieve electrosynthesis of value‐added chemicals, for example, acetate,^[^ ^4^ ^]^ biofuels,^[^ ^5^ ^]^ bioplastics,^[^ ^6^ ^]^ terpenes,^[^ ^7^ ^]^ and vitamins.^[^ ^8^ ^]^ However, for all these reactions, the low EET rate between EAMsprecursor and electrodes is often a major bottleneck for practical applications of BES. To increase the EET of EAMs, previous endeavors studied how cellular physiology impacted the electrophysiology of EAMs, such as substrate utilization,^[^ ^9^ ^]^ reducing equivalents (NAD^+^/H) precursor synthesis and regeneration,^[^ ^10^ ^]^ expression and maturation of *c*‐type cytochromes,^[^ ^11^ ^]^ assembly of conductive nanowires,^[^ ^12^ ^]^ synthesis and secretion of electron shuttles,^[^ ^13^ ^]^ and conductive biofilm formation.^[^ ^14^ ^]^

Cell dimension is an essential physiological function that has significant effects on cell growth and metabolism.^[^ ^15^ ^]^ Recently, a number of studies explored molecular and genetic mechanisms of change of cell dimension,^[^ ^16^ ^]^ and investigated its impact on cell metabolism, thus to enhance chemicals production.^[^ ^17^ ^]^ For example, Chen et al. increased the cell size of *Escherichia coli* (*E. coli*) by weak expression of the actin‐like protein MreB and overexpression of division inhibitor SulA, which resulted in 100% increase in accumulation of polyhydroxybutyrate (PHB) granules.^[^ ^18^ ^]^ Ding et al. applied an optogenetic system combining blue and near‐infrared light to dynamically control the cell size of *E. coli* by prolonging the C and D periods, which increased the production of poly(lactate‐*co*‐3‐hydroxybutyrate).^[^ ^17b^ ^]^ However, the relation between cell dimension and energy metabolism of cells was rarely investigated. Notably, a recent study showed that treatment of the EAM *Shewanella oneidensis* (*S. oneidensis*) MR‐1 with the cell‐division‐inhibitor cisplatin converted the rod‐shaped cells to elongated, filamentous forms,^[^ ^19^ ^]^ which increased the output current density from 111 ± 10.4 (*S. oneidensis* MR‐1) to 646 ± 40.9 mA m^−2^ (*S. oneidensis* MR‐1 with cisplatin treatment). Zhao et al.^[^ ^20^ ^]^ increased the cell length by overexpressing cell division inhibitor SulA, which enhanced biofilm thickness, but the specific underlying mechanism for manipulating cell length and its impact on cell electrophysiology has yet been systematically established.

**Figure 1:**
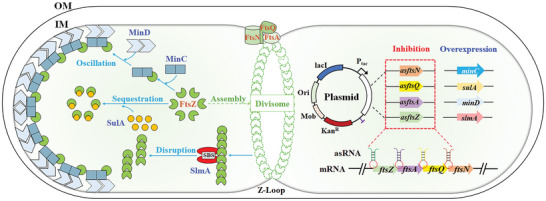
The design approach to increase cell length by inhibiting the divisome formation of *S. oneidensis*. The mechanism underlying the cell division of *S. oneidensis* can be divided into two modules: divisome assembly and divisome disruption. The divisome assembly module contained four proteins responsible for the divisome formation, including the protein FtsZ that formed Z‐loop at the mid cell by self‐polymerizing into filaments to localize and initiate cell division after DNA replication,^[^ ^21a^ ^]^ the cell membrane anchorin FtsA to recruit downstream division proteins FtsQ and FtsN, the recruiting and scaffolding protein FtsQ to control the assembly of early and late cell division proteins, and the trigger protein FtsN for septal peptidoglycan synthesis,^[^ ^21b^ ^]^ the translation of which were inhibited by asRNAs. The divisome disruption module contained four division inhibitors for disrupting the FtsZ filament, including the membrane‐linked Min system (MinC/MinD) for the oscillating FtsZ monomer to cell pole to prevent the formation of the Z ring away from the mid cell,^[^ ^24a^ ^]^ the nucleoid‐binding SlmA linked to the chromosome to prevent the formation of Z ring over the origin‐proximal region of the nucleoid by disrupting FtsZ polymer,^[^ ^24c^ ^]^ and the cytoplasmic SulA to sequester FtsZ monomer,^[^ ^24b^ ^]^ which were overexpressed to achieve cell elongation.

Bacterial cell length is essentially determined by relative rates of cell division, which is controlled by activities of divisome gene products,^[^ ^21^ ^]^ enabling the change of cell shape from rods to fibers or mini‐cells.^[^ ^15^, ^17^, ^18^, ^22^ ^]^ The programming of cell length in *S. oneidensis* MR‐1 was thus classified into two modules (**Figure** **1**), namely the divisome assembly module, and the divisome disruption module. In the divisome assembly module, four division proteins are responsible for the divisome formation: 1) the Z‐loop‐forming protein FtsZ, 2) the cell membrane anchorin protein FtsA, 3) the recruiting and scaffolding protein FtsQ, and 4) the trigger protein FtsN for septal peptidoglycan synthesis.^[^ ^21^ ^]^ Hence, downregulating the expression level of these division‐associated proteins to hinder divisome formation could potentially increase cell length.^[^ ^18^, ^23^ ^]^ In the divisome disruption module, four division inhibitors are responsible for disrupting the FtsZ filament: 1) two proteins involved with the membrane‐linked Min oscillation system (MinC/MinD), 2) a nucleoid‐associated division inhibitor SlmA, and 3) a cytoplasmic division inhibitor SulA that sequester FtsZ monomer or disrupt the FtsZ polymer.^[^ ^24^ ^]^ Upregulation any of these division inhibitors (MinC, MinD, SulA, or SlmA) may disrupt the divisome formation thus elongate cells.

In this study, the cellular length of *S. oneidensis* MR‐1 was engineered via inhibiting the formation of cell divisome (Figure 1). The electrophysiology of cell elongation was systematically investigated in terms of three biological mechanisms, namely intracellular electron generation, EET, and electroactive biofilm formation. Moreover, a quorum sensing (QS)‐based dynamic regulation circuit was integrated to separate cell growth and elongation phases, thus regained cell growth impaired by cell elongation. The cell length of the finally obtained strain was enormously increased, and output power density was enhanced to 3.41‐fold of that of WT with superior efficiency for pollutant treatment. We concluded that cell length plays an important role in determining cell electrophysiology, and engineering cellular length provides an innovate avenue to enhance the EET of EAMs.

## 2 Results

### 2.1 Program Cell Elongation by Inhibiting Divisome Formation

To increase cell length of *S. oneidensis* MR‐1, the genes *ftsA*, *ftsN*, *ftsQ*, and *ftsZ* encoding division proteins were individually deleted. However, deletions of these genes were found to be fatal to *S. oneidensis* MR‐1. Anti‐sense RNAs (asRNA)^[^ ^25^ ^]^ were thus designed to inhibit the translation of FtsA, FtsN, FtsQ, and FtsZ, which resulted in four recombinant strains AsftsA, AsftsN, AsftsQ, and AsftsZ (Figure 1, Tables S1 and S2, Supporting Information), respectively. Previous studies demonstrated that asRNA could not only function at the translational level but also repress gene transcription or trigger mRNA degradation,^[^ ^25^ ^]^ thus expression of the target genes was indeed repressed (Figure S1A, Supporting Information). Meanwhile, strains MinC, MinD, SlmA, and SulA were constructed for overexpressing the corresponding division inhibitor genes, respectively (Figure 1, Tables S1 and S2, Supporting Information), and the transcriptional levels of these target genes (*minC*, *minD*, *slmA*, and *sulA*) were increased (Figure S1B, Supporting Information).

**Figure 2:**
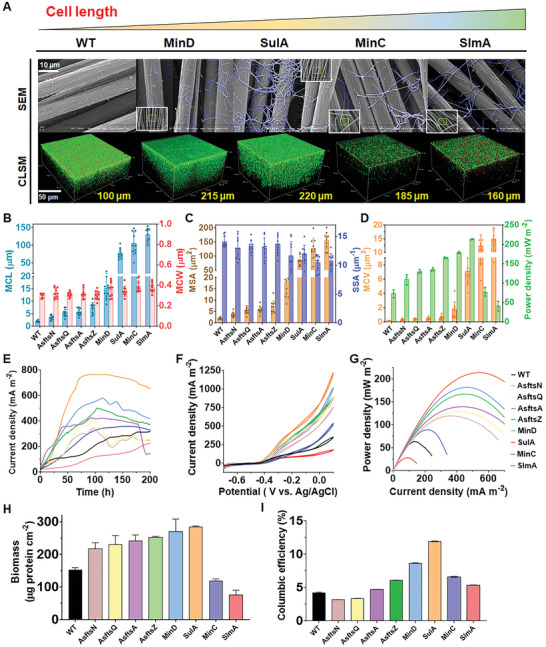
Morphological and electrochemical performance of the elongated strains. A) SEM and CLSM images of WT and four elongated strains overexpressing division inhibitors, ordered from left to right by increasing cell length (scale bar: 10 µm for SEM; 50 µm for CLSM images). Green fluorescence represented live cells labeled with SYTO 9, red fluorescence represented dead cells labeled with propidium iodide (PI). The excitation/emission wavelengths were 480/500 nm for SYTO 9 stain and 490/635 nm for PI. B) Mean cell length (MCL) and mean cell width (MCW) of WT and eight elongated strains. C) Mean surface area (MSA) and specific surface area (SSA) of WT and eight elongated strains. D) Mean cell volumes (MCV) and power densities of WT and eight elongated strains. E) Time–current curves of the MFCs incobated with WT and eight elongated strains. F) Cyclic voltammetry (CV) curves of the MFCs inoculated with WT and eight elongated strains under turnover conditions with a scan rate of 1 mV s^−1^. G) Power density curves of WT and eight elongated strains obtained by linear sweep voltammetry. H) Electrode‐attached biomass of WT and eight elongated strains after discharge. I) Columbic efficiencies of WT and eight elongated strains. Data were presented by three independent biological replicates as means ± SD.

Scanning electron microscopy (SEM) examination showed that all these eight strains were longer than the WT *S. oneidensis* MR‐1 (WT) (**Figure** **2**A; Figure S2, Supporting Information), confirming that inhibition of cell division could increase cell length. Particularly, cells of the strains SulA, MinD, MinC, and SlmA were filamentous and entangled on the anode, indicating the division inhibitors were more effective for cell elongation. The mean cell length (MCL) of the engineered strains all increased in comparison to that of WT (1.95 ± 0.33 µm), while the mean cell width (MCW) of these strains was close to that of WT (Figure 2B). Accordingly, based on the model of rod‐shape (Figure S3, Supporting Information; Equations (2)–(4), the quantified mean surface area (MSA) and mean cell volume (MCV) of the engineered strains were both increased (Figure 2C,D), while the specific surface area (SSA) was slightly decreased. Among these strains, strain SlmA was the longest, with a length of 130.0 ± 18.8 µm, which was 65.7‐fold higher than that of WT.

To determine the impact of cell elongation on EET, the output current density and power density of these strains were measured (Figure 2E–G). Compared to WT, the output current and maximum power output of the engineered strains AsftsN, AsftsQ, AsftsA, AsftsZ, MinD, and SulA increased in accordance with their MCV (Figure 2D). Correspondingly, they also displayed thicker biofilm, cell abundance on electrodes, and higher columbic efficiency in comparison to those of WT (Figure 2A,H,I; Figure S2, Supporting Information). Strain SulA, with cellular length of 77.3 ± 14.3 µm, achieved the highest power density of 212.9 ± 1.1 mW m^−2^, 3.2‐fold of that of WT (72.8 ± 10.1 mW m^−2^). Although strains MinC (105.2 ± 32.4 µm) and SlmA (130.0 ± 18.8 µm) were longer than strain SulA, their output power densities (76.6 ± 10.8 mW m^−2^ and 40.5 ± 12.5 mW m^−2^, respectively), biofilm thickness, cell abundance on electrodes, and columbic efficiency were not higher than strain SulA (Figure 2A,H,I; Figure S2, Supporting Information). Collectively, these results suggested that an increase in cell length could promote EET efficiency.

### 2.2 Electrophysiological Analyses of Cell Elongation

**Figure 3:**
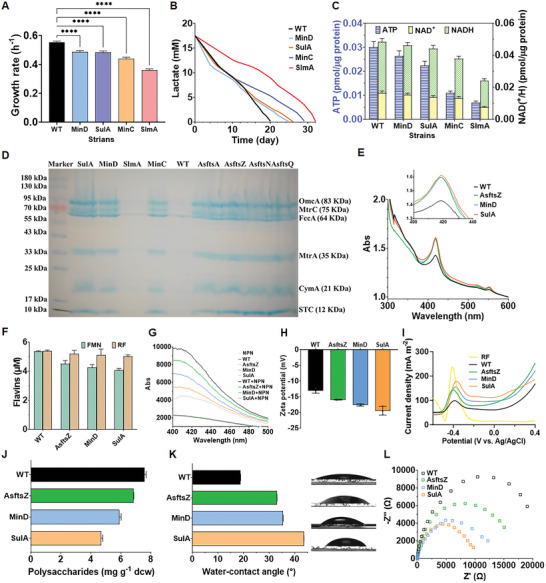
Electrophysiological performance of the elongated strains. A) Specific growth rates of the WT and the four elongated strains expressing the division inhibitors calculated by fitting growth curves with the Logistic curves using the GraphPad Prism 10.1.2 software. Significant difference was analyzed by the ordinary one‐way ANOVA method. B) Lactate consumption curves of WT and the four engineered strains expressing division inhibitors in MFCs. C) Intracellular ATP assay and NAD(^+^/H) measurement of the WT and the four elongated strains MinD, SulA, MinC, and SlmA. D) SDS‐page and heme staining of *c*‐type cytochromes of WT and eight elongated strains, which were normalized to OD_600 _= 0.5. E) UV/vis spectral characterization of total *c*‐Cyts levels of WT and the elongated strains AsftsZ, MinD, and SulA. Insert was the magnification of the section of 400–420 nm. Data are normalized to OD_600_ to account for cell density. F) Flavins concentration of the WT and elongated strains. G) Membrane permeability measured by NPN uptake assay. H) Zeta potentials of cells of the WT and elongated strains. I) Differential pulse voltammograms (DPV). J) Extracellular polysaccharide contents of the WT and elongated strains AsftsZ, MinD, and SulA. K) Water‐contact angles of strains WT, AsftsZ, MinD, and SulA. L) Electrochemical impedance spectra (EIS) analysis of strains WT, AsftsZ, MinD, and SulA in MFCs. Data were presented by three independent biological replicates as means ± SD. Significant difference was analyzed by the ordinary one‐way ANOVA method.

To investigate how increased cell length impacted intracellular electron generation, cell growth and metabolism of the programmed elongated strains were analyzed. Although the expression of division inhibitors was controlled at a minimum level to reduce their intrinsic cytotoxicity, the growth rate of strains MinD, SulA, MinC, and SlmA was severely declined (Figure S4, Supporting Information). The specific growth rates of strains MinD (0.486 ± 0.010 h^−1^), SulA (0.482 ± 0.011 h^−1^), MinC (0.439 ± 0.010 h^−1^) and SlmA (0.359 ± 0.011 h^−1^) were lower than that of WT (0.553 ± 0.008 h^−1^) (**Figure** **3**A; Figure S5A, Supporting Information), suggesting that the overexpression of these division inhibitors inhibited cell growth. The growth defects further impeded lactate uptake (Figure 3B), which consequently reduced the intracellular electron pool (namely the total level of NAD(^+^/H)) and metabolic activity (namely the ATP level) (Figure 3C) of strains MinD, SulA, MinC, and SlmA. On the other hand, the expression of asRNAs had a negligible effect on cell growth (Figure S5B, Supporting Information). Notably, the specific growth rate of the strains increased in the order of WT > AsftsN > AsftsQ > AsftsA > AsftsZ > MinD > SulA > MinC > SlmA. Correspondingly, the cell doubling time increased in the opposite order, that is, WT <AsftsN <AsftsQ <AsftsA <AsftsZ <MinD <SulA < MinC <SlmA (Figure S5C,D, Supporting Information; Equation (3)). These results showed that the specific growth rate of strains decreased along with cell length, while the doubling time increased along with cell length, which validated the “allometric scaling” phenomenon in prokaryotes,^[^ ^16^, ^26^ ^]^ namely “the smaller an organism, the faster the doubling time of its biomass”.

The levels of *c*‐cytochromes (*c*‐Cyts) in the constructed strains were also measured to determine the impact of cell elongation on direct EET. Heme staining, UV/vis spectroscopy, and immunofluorescence localization experiments consistently suggested that cell elongation increased *c*‐Cyts level. Specifically, heme staining revealed a complete Mtr conduit of *S. oneidensis*, including the inner membrane *c*‐Cyt CymA (21 kDa), the periplasmic *c*‐Cyts FccA (64 kDa) and STC (12 kDa), as well as the outer membrane *c*‐Cyts MtrA (35 kDa), MtrC (73 kDa), and OmcA (83 kDa) (Figure 3D). At OD_600 _= 0.5, the lane of these c‐Cyts in WT and SlmA were weak, but prominent in other strains especially AsftsZ, MinD, and SulA. UV/vis spectroscopy measurements consistently revealed the total *c*‐Cyts levels of strains AsftsZ, MinD, and SulA were elevated as compared with WT and SulA was the highest (Figure 3E). Immunofluorescence localization was performed to observe the distribution of outer membrane *c*‐Cyt MtrC in different morphologic strains (WT vs SulA). The more pronounced fluorescent signals on the surface of strain SulA indicated that the density of cell surface *c*‐Cyts of strain SulA was higher than that of WT (Figure S6, Supporting Information). Previous investigations have demonstrated the importance of *c*‐Cyts for direct contact‐based EET in nanometer distance.^[^ ^1^, ^11^, ^27^ ^]^ Thus, cell elongation was beneficial for direct EET.

To explore whether cell elongation changed flavins‐based EET, we first detected the flavins synthesis and secretion in the WT and elongated strains. As shown in Figure 3F,G, both the flavin concentrations and cell envelope permeability of the elongated strains were slightly decreased. Thus, the synthesis and secretion of riboflavin of elongated strains were both slightly decreased. However, the CV curves in Figure 2F suggested that the rise in bioelectric current occurred at ≈−0.4 V (vs Ag/AgCl), which apparently revealed that the flavins‐based EET was enhanced in the elongated strains. Previous studies showed that flavins could not only act as a reversible terminal electron acceptor to mediate indirect EET from the planktonic cells or biofilm to the electrode via diffusion,^[^ ^28^ ^]^ but also bind with outer‐membrane *c*‐Cyts MtrC and/or OmcA to facilitate the one‐electron redox reaction via forming semiquinone.^[^ ^29^ ^]^ Thus, to further validate this hypothesis, we performed Zeta potential measurements and differential pulse voltammetry (DPV) analyses. The results demonstrated that the Zeta potential of the surface of strain SulA was more negatively charged than that of the WT, which could enhance riboflavin absorption and binding (Figure 3H). Meanwhile, the DPV curve of the free riboflavin obtained an anodic peak at a redox potential of −0.41 V (Figure 3I). Notably, the observed redox peaks of the WT and the engineered strains shifted to −0.37 V, indicating the existence of cytochrome‐bound flavins peak.^[^ ^29a,c^ ^]^ Moreover, the peak height of the elongated strains AsftsZ, MinD, and SulA considerably surpassed that of the WT and increased in positive correlation with cell length. Specifically, the peak height of strain SulA was 1.85‐fold that of WT, suggesting that the flavins binding‐based EET was facilitated by cell elongation. Collectively, although the flavins synthesis and secretion were slightly decreased, the *c*‐Cyts‐flavins binding‐based one‐electron redox reaction was facilitated in the elongated strains.

Cell‐surface hydrophobicity and biofilm conductivity of the constructed strains were also measured to explore the impact of cell elongation on electroactive biofilm formation. Due to the decreased content of cell‐surface polysaccharides (Figure 3J), the water‐contact angles of strains AsftsZ (33.02 ± 0.06°), MinD (35.37 ± 0.17°), and SulA (43.53 ± 0.04°) were higher than that of WT (18.78 ± 0.04°) (Figure 3K), implying the surfaces of strains AsftsZ, MinD, and SulA were more hydrophobic than that of WT. Many studies have demonstrated that bacterial cell‐surface hydrophobicity plays an important role in promoting surface adhesion.^[^ ^30^ ^]^ The enhanced cellular hydrophobicity of the engineered *Shewanella* strains could promote cellular attachment on the hydrophobic carbon electrode and further aggregation of cells, which facilitated the initial surface adhesion process in the course of biofilm formation.^[^ ^31^ ^]^ Accordingly, confocal laser scanning microscopy (CLSM) observation showed that the biofilm thickness of strains AsftsZ (190 ± 9 µm), MinD (215 ± 7 µm), and SulA (220 ± 5 µm) were higher than that of WT (100 ± 4 µm), and increased in accordance with the increase in their cell hydrophobicity (Figure 2A; Figure S2, Supporting Information). The enhanced formation of electroactive biofilm consequently increased the direct contact‐based EET of strains.^[^ ^14^, ^32^ ^]^ Moreover, the internal charge‐transfer resistances of the biofilms formed by the strains AsftsZ (13.8 ± 2.5 kΩ), MinD (12.1 ± 1.6 kΩ), and SulA (8.3 ± 0.8 kΩ) were decreased compared with that of WT (22.6 ± 3.4 kΩ) (Figure 3L), suggesting increase in their biofilm conductivity. Furthermore, the layer of extracellular polymeric substances (EPS) in the biofilm of strain SulA (165 ± 14 µm) was thicker than WT (75.0 ± 9 µm) (Figure S7, Supporting Information), which could serve as media to assist EET associated with biofilm.^[^ ^13^, ^33^ ^]^ Since previous studies reported that Shewanella can secret *c*‐Cyts extracellularly,^[^ ^34^ ^]^ we performed UV/vis spectral characterization of the EPS from the WT and SulA strains (Figure S8, Supporting Information). The results showed that more *c*‐Cyts existed in the EPS of strain SulA than that in WT, thus enhanced biofilm conductivity.

### 2.3 Transcriptomic Analyses of Cell Elongation

**Figure 4:**
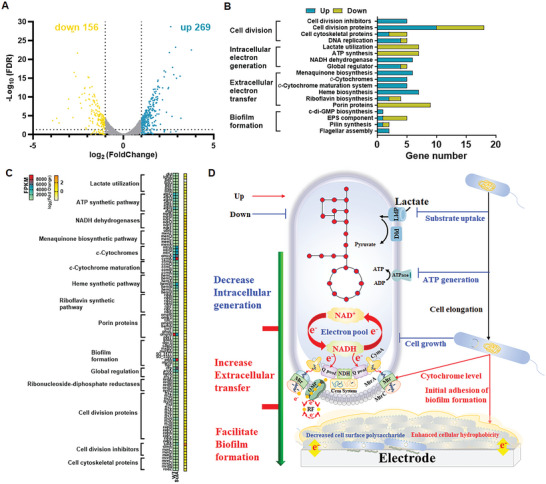
Transcriptomic analysis of cell elongation. A) Volcano plots for differential gene expression analysis. B) The number of upregulated and downregulated genes in different electrophysiological categories. C) Transcriptional levels of genes involved in lactate utilization, ATP synthetic pathway, NADH dehydrogenase, menaquinone synthetic pathway, *c*‐Cyts and maturation system, heme synthetic pathway, riboflavin synthetic pathway, biofilm formation, global regulation, porin proteins, ribonucleoside‐diphosphate reductases, cell division proteins, division inhibitors, and cytoskeletal proteins in the strains WT and SulA. D) Summary of the underlying mechanism of the enhanced electricity production. Cell elongation increased *c*‐Cyts‐based EET and facilitated electroactive biofilm formation but decreased intracellular electron generation. Data were presented by three independent biological replicates as means ± SD.

To investigate the specific mechanisms underlying enhanced power production, the transcriptomes of SulA and WT were comparatively analyzed (**Figure** **4**, Table S9, Supporting Information). RNA‐seq of strain SulA and WT generated a total of 4110 and 4121 genes reads that mapped to the reference genome (NZ_CP053946.1) with 99.54% and 99.81% coverage, respectively. Compared to that of WT, there were 269 genes that were upregulated and 156 genes that were downregulated (FDR <0.05, |log_2_FoldChange| > 1) in strain SulA (Figure 4A). Among them, apart from the genes involved in manipulating cell division, the main upregulated gene were distributed in NADH dehydrogenase, menaquinone synthetic, *c*‐Cyts expression and maturation, and heme synthetic pathways, while the genes for lactate utilization, ATP synthesis, and riboflavin transport were mostly downregulated (Figure 4B,C). Thus, the biological mechanisms underlying the reinforced EET in the elongated cells could be elucidated from three aspects: intracellular electron generation, direct electron transmembrane transfer, and electroactive biofilm formation, which were detailed in the following (Figure 4D):

- Cell elongation decreased the SSA and caused various physiological stress responses, including impaired transport of nutrients and decrease in metabolic activities responsible for cell proliferation, which led to inhibition of cell growth.^[^ ^15^, ^18^, ^35^ ^]^ Cell growth deceleration further disturbed celluar metabolism, thus decreasing intracellular electron generation. Expression of the genes encoding the lactate permease (*lldP*) and dehydrogenases (*ldhA* and *dld*) as well as the ATP synthetic genes *atpABEFGHI* was downregulated in strain SulA (Figure 4C, Table S9, Supporting Information). The source of the intracellular electron pool is generated from lactate catabolism. ATP biosynthesis is driven by both lactate‐based substrate‐level phosphorylation and proton motive force from NADH oxidation.^[^ ^36^ ^]^ We observed that the downregulation of these genes indeed resulted in impaired utilization of lactate and decreased levels of intracellular NAD(^+^/H) and ATP (Figure 3B,C).
- Cell elongation promoted direct electron transmembrane transfer by increasing NADH oxidation, inner‐membrane quinone pool, and *c*‐Cyts’ level. Notably, the genes *nqrABCDEF* encoding the type III NADH dehydrogenase and the genes *menACDEF* and *ubiE* involved in menaquinone (MQ) biosynthesis were upregulated in strain SulA, suggesting NADH oxidation was enhanced, and the inner‐membrane quinone pool was expanded. Moreover, the genes for the Mtr EET pathway (i.e., *mtrCAB*, *omcA*, and *cymA*), for heme biosynthesis (i.e., *hemCDEHNWH*), and for *c*‐Cyts maturation (*ccmABCDE*) were also upregulated in strain SulA, suggesting that both the expression and maturation of *c*‐Cyts were facilitated. CymA, MtrA, MtrC, and OmcA are multiheme *c*‐Cyts that transfer electrons from menaquinol (MQH_2_) in the cytoplasmic membrane, across the cell envelope to the extracellular electrodes. The transferred electrons from MQH_2_ were replenished via NADH oxidation by the type III NADH dehydrogenase.^[^ ^1^, ^37^ ^]^ The upregulation of the gene *cymA*, *mtrCAB*, and *omcA* also led to an increase in the level of *c*‐Cyts, as demonstrated by UV/vis spectroscopy, heme staining, and immunofluorescence localization experiments (Figure 3D,E; Figure S5, Supporting Information).
- Cell elongation promoted biofilm formation. Figure 3K showed that the surface of strain SulA was more hydrophobic than that of WT. Consistent with this observation, expression of the genes for biosynthesis of cell‐surface polysaccharide, such as *capD*, *mxdD*, *metXA*, and *SO_3177* was downregulated in strain SulA (Figure 4C, Table S9, Supporting Information). To be specific, the *SO_3177* gene was reported to encode a putative formyltransferase and situate in a cell surface polysaccharide biosynthesis gene cluster.^[^ ^31b^ ^]^ Similar, gene *mxdA* was also involved in a putative polysaccharide biosynthesis (EPS) gene cluster (*mxdABCD*).^[^ ^38^ ^]^ Downregulation of these genes could increase surface hydrophobicity, which facilitated the initial adhesion course of biofilm formation.^[^ ^30^, ^31^ ^]^ Moreover, the upregulated genes associated with biofilm formation included *bolA*, *dgcS*, *flgF*, *motB*, and *pilA* (Figure 4C, Table S9, Supporting Information). BolA is a transcription factor, and DgcS is a synthase for the second messenger c‐di‐GMP, which were identified to regulate biofilm formation.^[^ ^39^ ^]^ The genes *flgF* and *motB* are involved in the synthesis of the basal‐body and motor of flagella, which could serve as scaffolds to accommodate the arrangement of EPS, thus supporting biofilm formation.^[^ ^40^ ^]^ The gene *pilA* is responsible for type IV pilin synthesis, which could assist cell attachment on the electrode surface.^[^ ^41^ ^]^ Thus, the transcriptional response of these genes all contributed positively to biofilm formation. Indeed, the biofilm of strain SulA was thicker than that of WT (Figure 2A).

Collectively, transcriptomics, electrochemical, and biochemical experiments synergistically confirmed cell elongation reinforced EET by increasing NADH oxidation, inner‐membrane quinone pool, and *c*‐Cyts level, and facilitated electroactive biofilm formation by enhancing cellular hydrophobicity.

### 2.4 Quorum Sensing (QS)‐Based Dynamic Regulation to Regain Cell Growth Inhibited by Programmed Cell Elongation

**Figure 5:**
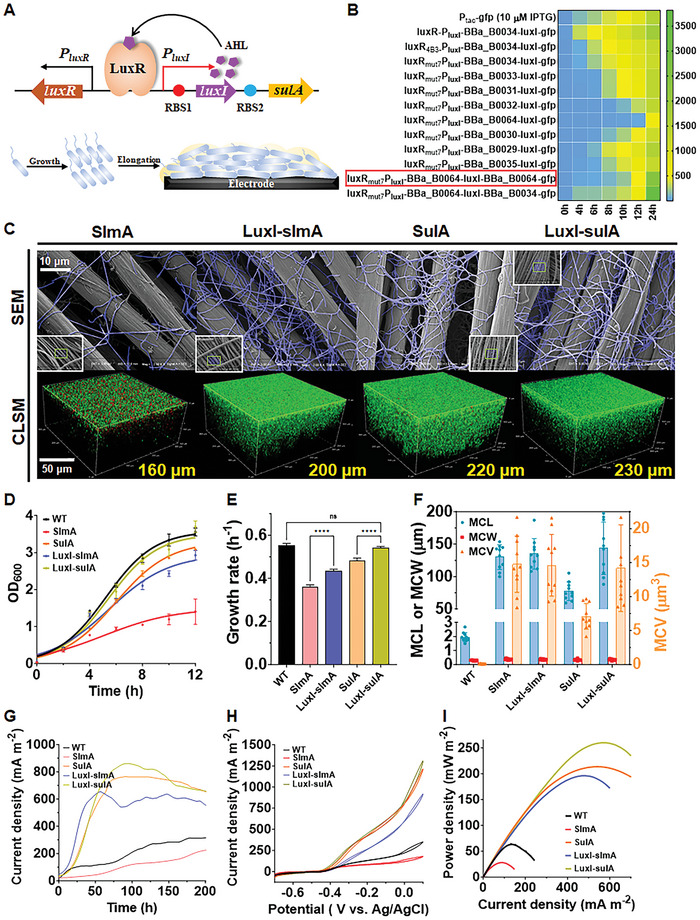
Quorum sensing (QS)‐based dynamic regulation to separate cell growth and elongation phases. A) Genetic design of the LuxR/LuxI‐based positive‐feedback‐loop QS circuit for dynamical control of cell elongation. B) Optimization of the QS system via protein mutagenesis of LuxR and RBS optimization (RBS1 for *luxI*; RBS2 for *gfp*), which enabled appropriate time for initiating cell elongation without causing detriment to cell viability. C) SEM and CLSM images of strains SulA, SlmA, and QS‐based strains LuxI‐sulA and LuxI‐slmA (scale bar: 10 µm for SEM; 50 µm for CLSM images). Green fluorescence represented live cells labeled with SYTO 9, red fluorescence represented dead cells labeled with propidium iodide (PI). The excitation/emission wavelengths were 480/500 nm for SYTO 9 stain and 490/635 nm for PI. D) Growth profiles of strains WT, SulA, SlmA, and QS‐based strains LuxI‐sulA and LuxI‐slmA cultured in LB medium, which were fitted with the Logistic curves by using the GraphPad Prism 10.1.2 software. E) Calculation of the specific growth rates of the WT, SulA, SlmA, and QS‐based strains LuxI‐sulA and LuxI‐slmA based on the simulated Logistic curves. Significant difference was analyzed by the ordinary one‐way ANOVA method. F) Mean cell length (MCL), mean cell width (MCW), and mean cell volume (MCV) of strains WT, SulA, SlmA, and QS‐based strains. G) Time–current curves of the WT and QS‐based strains during MFC cultivation. H) CV curves of MFCs inoculated with WT and strains LuxI‐slmA and LuxI‐sulA expressing quorum sensing system under turnover conditions with a scan rate of 1 mV s^−1^. I) Power density curves of strains WT, SulA, SlmA, and QS‐based strains based on linear sweep voltammetry analysis. Data were presented by three independent biological replicates as means ± SD. Significant difference was analyzed by the ordinary one‐way ANOVA method.

Although inhibiting cell division could enhance cellular length thus promoting direct contact‐based EET and biofilm formation, premature expression of the division inhibitors diminished cell growth, which in turn impaired intracellular electron generation. Quorum sensing (QS), a mechanism for cell–cell communication, could achieve autonomous regulation of cellular behavior in response to cell density.^[^ ^42^ ^]^ Thus, to eliminate growth deceleration caused by cell elongation, we designed a QS‐based positive‐feedback‐loop genetic circuit to dynamically control the expression of the division inhibitor (**Figure** **5**A). To be specific, this circuit included LuxR/LuxI, a widely used QS system from *Vibrio fischerii*,^[^ ^42^ ^]^ and a division inhibitor (i.e., SulA). LuxI produced the signal molecule acylated homoserine lactone (AHL), which bound to the transcription regulator LuxR to initiate the transcription of the promoter P_luxI_. Thus, at the early stage of cell growth, the AHL concentration is too low to activate downstream expression of division inhibitor, which would not lead to cell elongation. With the cell growth, the accumulated AHL would diffuse through the cell membrane to receiver cells and bind to LuxR to activate the promoter P_luxI_, which could facilitate the expression of LuxI to synthesize more AHLs in turn. Once the AHL concentration reached a certain threshold, the downstream expression driven by P_luxI_ would be triggered. Collectively, the expression of the downstream division inhibitor would be weak in the earlier stage and strong in the later period of cell growth. Thus, this QS‐based positive‐feedback‐loop circuit could separate the cell growth and elongation phases by delaying the expression of the division inhibitor SulA.

To achieve QS‐based tight and precise regulation of cell elongation while not causing detriment to cell growth, the initiation and duration time of the expression of the division inhibitor needed to be optimized in an appropriate manner. Since the cell elongation proceeded under aerobic conditions before deposition of cells for anaerobic MFC studies, an oxygen‐sensitive GFP‐based positive‐feedback‐loop genetic circuit (*luxR‐P_luxI_‐luxI‐gfp*) was first constructed to characterize the performance of the QS system in *S. oneidensis* MR‐1 (Figure S9A, Supporting Information). Real‐time quantitative reverse transcription PCR analyses confirmed the successful heterologous expression of both LuxR and LuxI in *S. oneidensis* MR‐1 (Figure S9B, Supporting Information). However, the GFP intensity under the dynamic regulation of the QS‐based positive‐feedback‐loop circuit was consistently higher than that under static regulation (the P_tac_ promoter) (Figure S9C, Supporting Information), which was caused by the high binding activity of LuxR on the promoter P_luxI_. To reduce the binding activity of LuxR on P_luxI_, we constructed two LuxR mutants, that is, LuxR_mut7_ and LuxR_4B3_,^[^ ^43^ ^]^ by the site‐directed mutagenesis method. Consequently, the GFP expression in the *luxR_mut7_‐P_luxI_‐luxI‐gfp* circuit showed a more sluggish response than that of the native LuxR (Figure S9C, Supporting Information). To further optimize the initial expression time of the LuxR_mut7_‐based circuit, nine pairs of ribosomal binding sites (i.e., RBS1 and RBS2) with different strengths quantified in *E. coli* by the International Genetically Engineered Machine Competition (iGEM), were designed to control the expression of *luxI* and *gfp*, respectively, in *S. oneidensis* MR‐1 (Table S7, Supporting Information). Among these constructs, the *luxR_mut7_‐P_luxI_‐BBa_B0064‐luxI‐BBa_B0064‐gfp* circuit exhibited the most ideal profile of the GFP expression, in which the GFP expression was weak at the initial stage, while increased substantially at the late growth stage of *S. oneidensis* MR‐1 (Figure 5B).

Strain SulA with the cell divisome module enabled the highest output power density but showed slight growth deceleration. We thus chose strain SulA to explore whether separating the cell growth and elongation phases could further enhance the cellular length and the EET rate. Meanwhile, strain SlmA processed the highest cellular length, but showed a siginificant decrease in growth rate. We thus chose strain SlmA to study whether relieving growth limitation could regain EET. Two QS‐based strains LuxI‐sulA and LuxI‐slmA were then constructed to dynamically regulate the expression of the division inhibitors by replacing the gene *gfp* in the *luxR_mut7_‐P_luxI_‐BBa_B0064‐luxI‐BBa_B0064‐gfp* circuit with the genes *sulA* and *slmA*, respectively (Tables S1 and S2, Supporting Information). Transcriptional analysis showed that the expression levels of the target genes in the QS‐based strains LuxI‐slmA and LuxI‐sulA drastically decreased in the early stage of cell growth (either at OD_600 _= 1 and 2 or 6 and 12 h) in comparison to that of the strains SulA and SlmA, respectively, while showed obvious increase at 24 h and surpassed that of the strains SulA and SlmA, respectively (Figure S9D–G, Supporting Information). These results synergistically suggested that the occurrence of cell elongation was successfully delayed by the QS‐based positive feedback loop circuits. SEM images showed the elongated cell morphology of strains SlmA and SulA, and the QS‐controlled strains LuxI‐slmA and LuxI‐sulA (Figure 5C). CLSM images showed that the biofilms of strains LuxI‐slmA (200 ± 6 µm) and LuxI‐sulA (230 ± 5 µm) were thicker than those of strains SlmA (160 ± 3 µm) and SulA (220 ± 5 µm) in the absence of the QS system, respectively (Figure 5C), indicating that QS‐based dynamic regulation enabled more filamentous cells entangling on electrodes.

Upon incorporating QS‐based regulation, the growth rate of the elongated strains recovered (Figure 5D). The specific growth rate of strain LuxI‐slmA (0.435 ± 0.007 h^−1^) exhibited 21.2% increase than that of strain SlmA (0.359 ± 0.011 h^−1^) (Figure 5E). In particular, the specific growth rate of strain LuxI‐sulA (0.542 ± 0.006 h^−1^) was close to that of WT (0.553 ± 0.008 h^−1^) without cytotoxicity (Figure S4I, Supporting Information), which suggested that QS‐regulated cell elongation could eliminate growth deceleration. Moreover, the lactate utilization rate, metabolic activity, and intracellular electron pool of strains LuxI‐slmA and LuxI‐sulA (Figure S10A,B, Supporting Information). MCL of strain LuxI‐sulA (143.6 ± 40.3 µm) further increased by 86% as compared to that of strain SulA, which was caused by the higher expression of the gene *sulA* under the QS regulation at the late growth stage. MCV of strain LuxI‐sulA was 14.2 ± 6.4 µm3, which was a twofold increase than that of SulA (Figure 5F). However, MCL (135.0 ± 23.5 µm) and MCV (14.6 ± 4.6 µm3) of strain LuxI‐slmA were similar to those of strain SlmA (Figure 5F). Accordingly, the output currents of the QS‐based strains further increased, and similar trends were observed in the output current densities in the LSV curves (Figure 5G–I). Specifically, the output power density of strain LuxI‐slmA increased to 187.5 ± 7.8 mW m^−2^ (sevenfold higher than strain SlmA), while strain LuxI‐sulA increased to 248.0 ± 10.6 mW m^−2^ (16.5% higher than strain SulA) (Figure 5I). Meanwhile, the anodic biomass and columbic efficiency all increased in comparison to those of strains SlmA or SulA, respectively (Figure S10C,D, Supporting Information). Furthermore, the *c*‐Cyts abundance of strain LuxI‐sulA was higher than that of strain SulA (Figure S10E, Supporting Information). Finally, the biofilms of strains LuxI‐sulA and LuxI‐slmA were also more conductive than those of strains SulA and SlmA, with internal resistance of 7.12 ± 0.31 and 9.98 ± 1.67 kΩ, respectively (Figure S10F, Supporting Information). Notably, the individual expression of the QS system in the normal‐size strain (LuxR) showed negligible effect on EET, lactate uptake, as well as intracellular ATP and NADH/NAD^+^ levels compared with those of WT (Figure S11A–D, Supporting Information), suggesting the enhanced EET was intrinsically caused by cell elongation rather than QS system per se. Collectively, all these results consistently demonstrated that the introduction of a QS‐based genetic circuit separated the cell growth and elongation phases, thus eliminating cell growth inhibition caused by cell elongation, which further increased cellular length and EET efficiency. Furthermore, the increased EET of the engineered strain LuxI‐sulA was also observed in other assays, such as Cr^6+^ reduction and azo dyes degradation experiments (Figure S12, Supporting Information).

## 3 Discussion

Many synthetic biology and material engineering strategies have been implemented to enhance the EET of exoelectrogens for power production by promoting intracellular electron generation, EET, and abiotic/biotic interfacial electron exchange.^[^ ^14^, ^44^ ^]^ However, how cell length impacts electrophysiology, power generation, and the underlying mechanisms has not been well studied. A previous study.^[^ ^20^ ^]^ constructed *S. oneidensis*‐materials hybrid biofilm comprising riboflavin, multiwalled carbon nanotubes, and graphene oxide (GO) with elongated *S. oneidensis* by solo overexpression of the *sulA* gene, to reduce the charge‐transfer resistance between the cells and electrode, however, the specific underlying mechanism for the enhanced biofilm formation was not systematically investigated. Conversely, the essential purpose of this study was to design and construct synthetic biology strategies to increase cell length and thoroughly elucidate the underlying mechanism how cell elongation impacts *c*‐Cyts‐based direct EET and flavins‐mediated indirect EET, intracellular electron generation and biofilm formation, as well as the resulting power output.

The culture environment and conditions.^[^ ^45^ ^]^ and perturbations of metabolic nodes.^[^ ^15^, ^35^ ^]^ have been reported to influence the morphology of *Shewanella*. In this study, to investigate the relationship between cell length and EET capability of *S. oneidensis* MR‐1, cell division was initially engineered by manipulating the formation of cell divisome, which essentially differed from previous studies. The cell length was increased by downregulating the expression of division proteins or overexpressing the cell division inhibitors. Consequently, cell elongation enhanced *c*‐Cyts level to promote direct contact‐based EET, and elevated cell‐surface hydrophobicity to facilitate initial adhesion in the course of biofilm formation, resulting in an output power density of 212.9 ± 1.1 mW m^−2^. However, the engineered cell elongation reduced cell growth due to division inhibition. Then, a QS‐based gene circuit (*luxR‐luxI‐sulA*) was designed to separate cell growth and elongation phases, thereby regaining cell growth and metabolism impaired by cell elongation, which increased the output power density to 248.0 ± 10.6 mW m^−2^, 3.4‐fold of WT *S. oneidensis* MR‐1. When normalized to the electrode‐attached biomass, the normalized power densities of the elongated strains (AsftsZ, MinD, SulA, as well as the QS‐based strains LuxI‐sulA and LuxI‐slmA) remained higher than that of WT, and increased in positive correlation with their cellular length (Figure S13, Supporting Information). This suggested that the elevated EET was not only caused by more anodic biomass on electrodes but also higher cellular electroactivity due to enhanced levels of *c*‐Cyts on the cell membrane enabled by cell elongation.

The best bio‐electrochemical performance of the strain SulA was caused by the synergistic effects of enhanced EET efficiency and cell metabolism. Cell elongation facilitated *c*‐Cyts expression and biofilm formation. We observed that the *c*‐Cyts expression and biofilm formation increased along with the cell length. The higher *c*‐Cyts level and cellular hydrophobicity of strain SulA endowed it with higher output power density than that of strains AsftsZ and MinD. On the other hand, when the cell length reached a certain threshold, it impaired cell growth and metabolism, which decreased intracellular ATP and NAD^+^/H levels, ultimately reducing *c*‐Cyts expression and biofilm formation of strains MinC and SlmA.

The use of quorum sensing (QS) approach to regulate the SulA expression was one of the key innovations of our study, which allowed us to effectively decouple the cell growth from cell elongation. We constructed the QS‐based gene circuit to ensure that the SulA expression was tightly controlled and only activated at a specific cell density. This study highlighted the potential of QS‐based gene circuits in achieving automatous and precise regulation of gene expression, which is of value in MFC optimization and other biotechnological applications.

Collectively, our study provided valuable insights into how cell elongation impacts the electrophysiology of *S. oneidensis* in terms of intracellular electron generation, EET, and electroactive biofilm formation. The specific underlying mechanisms were elucidated as three aspects (see Figure 4D): i) cell elongation impeded cell growth and lactate uptake, which decreased intracellular electron generation. We thus designed a QS‐based dynamic regulation gene circuit to retrieve cell growth, thus regain intracellular electron pool; ii) cell elongation reinforced direct EET by enhancing *c*‐Cyts level on the cell membrane. Additionally, the increased *c*‐Cyts further facilitated high‐efficient one‐electron redox reaction through *c*‐Cyts‐bound flavins; and iii) cell elongation facilitated electroactive biofilm formation by enhancing cellular hydrophobicity. Thus, an increase in cell length was a feasible approach to promote the EET of EAMs.

## 4 Experimental Section

### Setup of Bio‐Electrochemical System

One milliliter of overnight cultures of the engineered strains of *S. oneidensis* MR‐1 suspension were separately added into 100 ml fresh LB broth with corresponding antibiotics and optimized IPTG. The cultures were incubated at 30 °C with shaking (200 rpm) for 12 h. Then, each culture was mixed with electrolytes (5% LB broth, 95% M9 buffers, 18 mm sodium lactate, antibiotics, and IPTG) to obtain a final optical density (OD_600_) of 0.5. Dual‐chamber MFCs (120 ml working volume) separated by nafion 117 membranes (DuPont Inc., USA) were used. A carbon cloth (CeTech WOS1002, from Rocktek, China) was used as the working electrode (1 cm × 1 cm) and counter electrode (2.5 cm × 3.0 cm). The cathodic electrolyte was composed of 50 mm K_3_[Fe(CN)_6_], 50 mm K_2_HPO_4_, and 50 mm KH_2_PO_4_. A 2 kΩ external resistor was connected into the external circuit of MFC, and the output current was recorded by a digital multimeter (DT9205A) every 0.5 h.

### Electrochemical Characterization

When the output current reached peak and stayed stable, cyclic voltammetry (CV) analysis with a scan rate (1 mV s^−1^) was performed in a two‐chamber three‐electrode configuration with an Ag/AgCl as reference electrode on a CHI1000C multichannel potentiostat (Chenhua Instrument, Shanghai, China). Then, linear sweep voltammetry (LSV) analysis with a slow scan rate (0.1 mV s^−1^) was conducted on a two‐electrode mode to estimate the maximum power density (the potential decreased from the open circuit potential −0.8 to −0.1 V). All the current density and power density were normalized to anode carbon cloth area of 1 cm^2^. Power density (*P*) was calculated using Equation (1):

(1) $$P=\frac{VI}{S}$$

where *V* is the output voltage; *I* is the current; and S is the projected area of the anode surfaces.

Electrochemical impedance spectroscopy was performed in a three‐electrode configuration with Ag/AgCl as the reference electrode and measured by MULTI AUTOLAB M204 (Metrohm, Netherlands) over the frequency range of 10^−2^–10^5^ Hz with 10 mV amplitude. The internal charge‐transfer resistance of each strain was calculated as the diameter of the semicircle at a medium‐high frequency of the Nyquist plot fitted by the Nova 2.1.4 software. For chronoamperometry, a constant potential of +0.2 V (vs Ag/AgCl) was added directly to anodic carbon cloth in a three‐electrode configuration. Differential pulse voltammetry (DPV) analysis was performed in a one‐chamber three‐electrode configuration on a CHI660e Electrochemical Analyzer/Workstation (Chenhua Instrument, Shanghai, China). Carbon cloth was used as the anode (1 cm × 1 cm), Ag/AgCl as the reference electrode, and platinum net (area of 4 cm^2^) as the counter electrode. DPV was performed with a scan window of −0.6 to 0.4 V, 1 mV s^−1^ scan rate, 1 s period, 0.5 s pulse time, 50 mV pulse size, and 1 mV step size.

### SEM Imaging of Cell Morphology

The morphology of the anodic cells of WT and engineered strains were characterized by a Field Emission Scanning Electron Microscopy (Tescan Mira LMS, Czech) with an acceleration voltage of 5 kV. Before observation, anodes were removed from operating MFCs and fixed in 2.5% glutaraldehyde for 3 h, then were dehydrated in a series of ethanol solutions (30%, 50%, 70%, 80%, and 90%) subsequently dried in a vacuum. Samples were divided into small pieces and coated with Au prior to the SEM analyses. The analyses were carried out in Shiyanjia Lab (https://www.shiyanjia.com/).

### Confocal Imaging of Biofilm, EPS, and Cell Viability

Bacterial biofilms were analyzed with a CLSM (Nikon A1R+). The carbon cloth electrodes after discharge were cut up and were washed in PBS (pH 6.8). Then the anodic biofilms were stained with the LIVE/DEAD BacLight bacterial viability kit (Invitrogen, USA) including SYTO 9 for live cells and propidium iodide (PI) for dead cells, respectively, and were scanned by the Laster with a step of 5 µm. The excitation/emission wavelengths were 480/500 nm for SYTO 9 stain and 490/635 nm for PI. For EPS observation, Fluorescein isothiocy anate (FITC), Concanavalin (ConA), Calcofluor white (CW), and PI were used for staining the extracellular protein, *α*‐polysaccharide, *β*‐polysaccharide, and extracellular DNA (eDNA), respectively. The obtained images were further processed by NIS‐Elements AR Analysis software.

### Measurement of Cellular Length and Width and Other Calculations

The length and width of a single cell were measured using ImageJ. For each strain, 10 individual cells were measured for calculating its MCL and MCW). MCV, MSA, and SSA (i.e., namely the ratio of cell surface area to volume) were calculated based on a cell rod‐shape model (Figure S3, Supporting Information) and the calculation formulas, Equations (2)–(4):

(2) $$S=4\pi {\left(\frac{D}{2}\right)}^{2}+\pi D\left(L-D\right)=\pi LD$$

(3) $$V=\frac{4}{3}\pi {\left(\frac{D}{2}\right)}^{3}+\pi {\left(\frac{D}{2}\right)}^{2}\left(L-D\right)=\frac{\pi }{12}\left(3LD^{2}-D^{3}\right)$$

(4) $$SSA=\frac{12LD}{3LD^{2}-D^{3}}=\frac{1}{D}\times \frac{12}{3-\frac{D}{L}}$$

where *L* is the cell length, *D* is the cell width.

The doubling time (*t*_d_) of the elongated cells could be calculated based on Equation (5)

(5) $$\mu =\frac{\ln2}{t_{d}}$$

where *µ* (h^−1^) is the specific growth rate calculated by fitting with the Logistic curves using the Origin2022 software.

### SDS‐Page and Heme Staining of *c*‐Cyts

Two milliliters overnight culture was collected by centrifugation (12 000 rpm for 1 min). After wash with sterilized 10 mm PBS buffer (pH 7.2–7.4) three times. The total membrane proteins of the engineered strains were extracted by the Bacterial Membrane Protein Extraction kit (Catalog No: PH1461, Phygene Life Sciences). The cells and purified membrane proteins were resuspended in 160 µL of distilled water and solving solution, respectively, with 40 µl of 5 × loading dye. The loading dye consisted of 0.25 m Tris HCl, pH 6.8, 50% (v/v) glycerol, 10% (w/v) SDS, and 0.5% (w/v) bromophenol blue. The resuspension was treated at 96 °C in a hot water bath for 10 min. After cooling to room temperature, 20 µl of the suspended cells was loaded on 12% (w/v) polyacrylamide gels for electrophoresis and then ran at 150 V for ≈1 h on a Bio‐Rad Mini‐PROTEAN Tetra Cell System. The electrophoresis running buffer contained 3.03 g L^−1^ Tris, 18.8 g L^−1^ glycine, and 1 g L^−1^ SDS. After electrophoresis, the gels were immersed in a solution that contained 30 mL 6.3 mm 3, 3′, 5, 5′‐tetramethylbenzidine (TMBZ) dissolved in methanol and 70 mL 0.25 m sodium acetate (pH 5.0). After incubating overnight in the dark and 4 °C, 3 mL 30% hydrogen peroxide was added and staining was visible within 30 min.^[^ ^46^ ^]^ The stained gels were imaged by Imagelab (Bio‐Rad) software.

### UV/Vis Spectrophotometer Measurement of *c*‐Cyts

Two ml cultures were collected by centrifugation (12 000 rpm, 5 min). After the removal of the supernatant, the cell pellets were resuspended in 10 mm PBS. The resuspended cells were lysed by ultrasonication in an ice bath (200 W, ultrasonicating‐2 s‐waiting‐1 s cycle for 1 min) with an Ultrasonic Homogenizer (Scientz, China). The *c*‐Cyts in cell lysate was measured using the UV/vis spectrophotometer (UV‐2450, Shimadzu). Oxidized *c*‐Cyts had a maximal Soret (*γ*) absorption peak at 408 nm, a visible peak at 531 nm, and a shoulder at 560 nm. Sodium dithionite was used to reduce *c*‐Cyts. Upon reduction, the Soret band maximum shifts to 419 nm, and the characteristic β‐and α‐Soret peaks became more prominent at 523 and 552 nm, respectively.

### Water‐Contact Angle Measurement

After 12 h of incubation, cells were harvested by centrifuging at 4000 rpm for 10 min. The cells were washed twice and suspended with PBS. Then suspension was filtered through a cellulose acetate membrane filter (0.45 µm). The filters were air‐dried at room temperature for 30 min. The contact angles were determined with the sessile drop method and a Ramé Hart 290‐F1 automated goniometer.^[^ ^31a^ ^]^ A drop of 2 µl of the water was dispensed on the surface of the filter, and contact angles were taken 2 s after drop deposition.

### Flavins Detection

Overnight cultures (2 mL) were collected by centrifugation (12 000 rpm, 1 min). The supernatants were used for flavin detection. All solutions and samples were filtered and assayed by the Alliance HPLC system [reverse‐phase C18 column (Symmetry, 5 µm, 4.6 × 250 mm), Waters e2695 separation module and 2998 PDA detector (Waters, USA)]. The mobile phase was composed of methanol and 0.01 m NaH_2_PO_4_ in a fixed ratio of 30: 70. The chromatographic separations utilized an elution rate of 0.6 ml min^−1^ and an injection volume of 20 µL within 25 min at 30 °C. Flavin concentrations were calculated based on the signals at 445 nm.

### Assessment of Cell Membrane Permeability

The overnight culture of cells was diluted by PBS buffer at a final OD_600_ of 0.5. The *N*‐phenyl‐1‐naphthylamine was then added into the cell suspension at a final concentration of 6 µm. After incubation for 10 min at 30 °C, the microplate reader (Tecan infinite 200 pro) was used to qualify the fluorescence emission (scanning from 400 to 500 nm) with the excitation at 355 nm.

### Zeta Potential Measurement

The cells were separated from the anode and washed thrice and then resuspended into ddH_2_O at a final OD_600_ of 0.1. The sample was then subjected to zeta potential measurement using a Zeta‐sizer Nano ZS system (Malvern, UK).

## Conflict Of Interest

The authors declare no conflict of interest.

## Author contributions

F.Li. and H.Y. contributed equally to this work. F.Li., H.Y., B.Z., and H.S. conceived and designed the research. H.Y., C.H., F.Lan., and Y.W. performed the experiments. Q.L., J.Z., and C.L. helped perform some experiments. F.Li., H.Y., B.Z., and H.S. analyzed the data. F.Li. and H.Y. wrote the manuscript. L.S., W.‐W.L., K.H.N., Z.L., and H.S. critically revised the manuscript. B.Z., Z.Y., and R.T. helped revise the manuscript. H.S. supervised the project.

## Supporting information



Supporting Information

## Data Availability

The data that support the findings of this study are available from the corresponding author upon reasonable request.
