# Supplementary material for: Engineered Cell Elongation Promotes Extracellular Electron Transfer of Shewanella Oneidensis
Source: Adv Sci (Weinh). 2024 Sep 5;11(41):2403067. doi: 10.1002/advs.202403067 (PMC11538702; doi:10.1002/advs.202403067)
Supplement: Supplementary file 1 — Supporting Information [file ADVS-11-2403067-s001.docx]

***Supplementary Information for***

**Engineered cell elongation promotes extracellular electron transfer of *Shewanella oneidensis***

Feng Li^1, ‡^, Huan Yu^1, ‡^, Baocai Zhang^1^, Chaoning Hu^1^, Fei Lan^1^, Yuxuan Wang^1^, Zixuan You^1^, Qijing Liu^1^, Rui Tang^1^, Junqi Zhang^1^, Chao Li^1^, Liang Shi^2^, Wen-Wei Li^3^, Kenneth H. Nealson^4^, ZhanYing Liu^5, *^, and Hao Song^1, 6, *^

^1^ Frontier Science Center for Synthetic Biology (Ministry of Education), Key Laboratory of Systems Bioengineering, and School of Chemical Engineering and Technology, Tianjin University, Tianjin, 300072, China.

^2^ Department of Biological Sciences and Technology, School of Environmental Studies, China University of Geoscience in Wuhan, Wuhan, Hubei 430074, China.

^3^ Chinese Academy of Sciences Key Laboratory of Urban Pollutant Conversion, Department of Environmental Science and Engineering, University of Science & Technology of China, Hefei 230026, China.

^4^ Departments of Earth Science & Biological Sciences, University of Southern California, 4953 Harriman Ave., South Pasadena, CA 91030, USA.

^5^ Center for Energy Conservation and Emission Reduction in Fermentation Industry in Inner Mongolia, Engineering Research Center of Inner Mongolia for Green Manufacturing in Bio-fermentation Industry, and School of Chemical Engineering, Inner Mongolia University of Technology, Hohhot, 010051, Inner Mongolia, China.

^6^ Haihe Laboratory of Sustainable Chemical Transformations, Tianjin, 300192, China

^‡^ These authors contribute equally to this work.

* Corresponding authors: ZY. Liu ([hgxylzy2008@imut.edu.cn](mailto:hgxylzy2008@imut.edu.cn)) and H. Song (hsong@tju.edu.cn)

The supplementary information includes:

- Supplementary methods
- Supplementary Tables 1-9
- Supplementary Figures 1-13

**Supplementary Methods**

**Genes synthesis, plasmids construction, and bacterial culture**

The specific mechanisms of genes that were utilized to program cell dimension, quorum sensing, and global regulation in this study were described in **Table S3**. All of the plasmids were by Gibson Assembly^[1]^ or DNA ligation. The genes *minC*, *minD*, *slmA*, and *sulA* were PCR amplified individually from the genome of *Shewanella oneidensis* MR-1 and cloned into a Biobrick compatible expression vector pYYDT by Gibson Assembly, obtaining plasmids pYYDT-minC, pYYDT-minD, pYYDT-slmA, and pYYDT-sulA, respectively. AsRNAs were designed as inverse complementary sequence of 100 bp of target genes (50 bp in the translation initiation region covering the RBS and 50 bp in the upstream of coding sequence) with its scaffold (PT template comprising two 38-bp inverted repeat DNA sequences to generate stem-loop structure^[2]^). The specific sequences of asRNAs were listed in **Table S4,** and were synthesized by Genewiz (Suzhou, China), then ligated in pYYDT through adding the upstream NdeI and downstream SalI restriction enzyme sites, obtaining plasmids pYYDT-asftsA, pYYDT-asftsN, pYYDT-asftsQ, and pYYDT-asftsZ. The coding sequences of the genes *luxR*, *luxR_mut 7_*, *luxR_4B3_*, and *luxI* from *Vibrio fischerii* were searched from NCBI database and adapted for expression in *S. oneidensis* MR-1 (<http://www.jcat.de/>), added with a ribosome binding site (RBS) and *gfp*, which were synthesized by Genewiz (Suzhou, China) and ligated in pYYDT through the BamHI and XhoI restriction enzyme sites, obtaining the plasmids pYYDT-luxR-gfp, pYYDT-luxR_4B3_-gfp, and pYYDT-luxR_mut7_-gfp. The plasmids pYYDT-luxR_mut7_-slmA and pYYDT-luxR_mut7_-sulA were constructed by replacing the *gfp* fragment of pYYDT-luxR_mut7_-gfp with the gene *slmA* and *sulA*, respectively. The primers used for plasmid construction were listed in **Table S5**. RBSs with different strengths measured in *Escherichia coli* by International Genetically Engineered Machine Competition (iGEM) (<http://parts.igem.org/Ribosome_Binding_Sites/Prokaryotic/Constitutive/Community_Collection>) were used to regulate the translation levels in the QS-based genetic circuits. The sequences of promoters and RBSs were listed in **Table S6** and **S7**.

Except for the QS-based regulation, each gene was controlled by an inducible *P_tac_* promoter with an optimized IPTG concentration. The IPTG was used at 10 µM for division inhibitor encoding genes and 1 mM for asRNAs, global regulator CRP encoding gene, cAMP synthetic genes and riboflavin biosynthesis genes. All constructed plasmids were first transformed in the auxotrophic donor strain *E. coli* WM3064. The resulting strains were cultured in Luria-Bertani (LB) medium supplied with 0.059 g l^-1^ 2,6-diaminopimelic acid (DAP) and corresponding antibiotics for plasmid maintenance at 37 °C, 220 rpm. Plasmids were then transferred into *S. oneidensis* MR-1 (ATCC700550) by conjugation. The resulting strains of *S. oneidensis* was cultured in LB medium with antibiotic and inducer IPTG at 30 °C, 200 rpm. The antibiotics were used at 50 μg ml^-1^ for kanamycin and 25 μg ml^-1^ for chloramphenicol. **Tables S1** and **S2** listed the strains and plasmids used in this study, respectively.

**Electrode-attached biomass measurements**

To determine the protein content of biofilm on electrode, carbon cloth anodes after MFC operation were placed in a 10 ml tube containing 3 ml PBS. The tube was vortexed for 2 min and then incubated in a water bath (at 96 °C for 20 min) to lyse cells. After cooling to room temperature, the extracts were analyzed using a bicinchoninic acid protein assay kit (Boster AR0146, China) according to the manufacturer’s instructions with 1 mg ml^-1^ bovine serum albumin (BSA) as standard.

**Extraction of EPS and qualification of polysaccharides**

40 mL of cell culture was harvested and suspended in 0.9% NaCl solution, and then were centrifuged with 5000 ×g for 10 min at 4 °C. The cell pellets were collected and washed by 0.9% NaCl solution for twice, incubated in water bath at 40 °C for 30 min, and then centrifuged with 5000 ×g for 15 min at 4 °C. Subsequently, the supernatants were filtered through 0.22 μm polytetrafluoroethylene membrane to obtain EPS. The residual cell precipitates were dried for measurement of dry cell weight (DCW). The concentrations of polysaccharides were quantified through the Anthrone-Sulfuric acid colorimetry^[3]^ and normalized to DCW.

**Transcriptome analysis**

Cells in WT, SulA, SD, and SDC-based MFC were harvested by centrifugation at 5000 ×g for 5 min at 4 ℃ and then flash frozen by liquid nitrogen. The cDNA / DNA / Small RNA libraries were sequenced on the Illumina sequencing platform by Genedenovo Biotechnology Co., Ltd (Guangzhou, China).

**Fluorescence measurement**

*S. oneidensis* MR-1 harboring plasmid with gene *gfp* was pre-inoculated into 5 ml LB with 50 μg ml^-1^ kanamycin and then cultured at 30 °C. Every two hours, 200 μl of the culture was transferred into a 1.5 ml EP tube and centrifuged at 12,000 rpm for 1 min. After the supernatant was removed the pellet was resuspended with 200 μl PBS. The resuspended cells were transferred into a 96-well microplate. The plate was placed in a microplate reader (Tecan infinite 200 pro). GFP fluorescence was measured using an excitation wavelength of 470 nm and an emission wavelength of 510 nm. The gain value was fixed to 50. GFP fluorescence value was normalized as GFP/OD_600_, and the background fluorescence of medium was subtracted.

**Immunofluorescence localization of MtrC**

Immunofluorescence localization was utilized for specific imaging of cell surface MtrC. The mouse-derived monoclonal antibody specific to MtrC was synthetized by Wuhan Genecreate Biological Engineering Go. Ltd (China). FITC Conjugated Affinipure Goat Anti-Mouse IgG (Boster, Catalog BA1101) was used as secondary antibody. The immune hybridization and fluorescence observation were carried out by Shiyanjia Lab (https://www.shiyanjia.com/).

**Quantification of the intracellular NAD(^+^/H)**

NAD^+^/H concentrations were measured using a NAD(H) content assay kit (Solarbio^®^ BC0300, China) according to manufacturer’s instructions. *S. oneidensis* MR-1 strains under MFC condition was sampled for NAD^+^/H quantification. 500 μl of cells were collected and immediately resuspended in 0.5 ml of acid extract (for NAD^+^) or alkaline extract (for NADH) by ultrasonication in ice bath (200W, ultrasonicating-2 s-waiting-1 s cycle for 1 min) with an Ultrasonic Homogenizer (Scientz, China). The cell lysate was further boiled for 5 min, cooled in ice bath, and centrifugated at 10,000 ×g at 4 °C for 10 min to remove cell debris. The supernatant was added with 500 μl of alkaline extract (for NAD^+^) or acid extract (for NADH). Visible spectrophotometry was used to determine the NAD^+^/H concentration by measuring the absorbance at 570 nm with 1.25 nmol/ml NAD and NADH as standards. The intracellular NAD^+^/H concentration were normalized to biomass measured by BCA assay kit.

**ATP assay**

ATP levels were measured using a visible spectrophotometry-based ATP content assay kit (Solarbio^®^ BC0300, China) according to manufacturer’s instructions. *S. oneidensis* MR-1 strains were cultured in under 30 °C and 200 rpm for 12 h. One ml of cultures was collected by centrifugation (12,000 rpm, 5 min) and resuspended in ATP detection lysis buffer for ultrasonication in ice bath. The cell lysate was further centrifugated 10000 ×g for 4 min at 4 ℃ and supernatant was collected. Then, 500 µl chloroform was added and mixed. After centrifugation, the supernatant was used for ATP detection with 0.0625 µmol/ml ATP as standard. The final ATP concentration were normalized to biomass measured by BCA assay kit.

**Lactate measurement**

A sterile syringe was used to take 1ml of culture solution in each MFC every 24 hours. After centrifugation at 12000 rpm for 1min, the supernatant was diluted between 1-100 mg ml^-1^ of lactate. Biosensor analyzer (SBA-40E) was used to measure the lactate concentration with 50 mg ml^-1^ lactate as standard.

**Columbic efficiency calculation**

The Coulombic efficiency (CE) is defined as the ratio of the actual Coulombs recovered as current to maximum possible Coulombs if all substrate was consumed to produce current. The Coulombs actually recovered was determined by integrating the current (I) over a period of batch cycles (t) ^[4]^. Thus, the Coulombic efficiency can be evaluated over a period of discharging using Eq. (6):

$$C_{E}=\frac{Coulombs recovered}{Total coulombs in substrate}=\frac{\int_{0}^{t_{b}} Idt}{Fb_{ES}V_{An}\Delta c} (6)$$

where F is Faraday’s constant (96487 C mol^−1^ of electrons), I (A) is the current, t_b_ (s) is the time period of a batch cycle, b_ES_ is the stoichiometric number of moles of electrons produced per mole of substrate (b = 4 when lactate was used as the substrate), V (l) is the volume of liquid in the anode compartment, and Δc (mol l^−1^) is the change of substrate concentration over a batch MFC cycle.

**Real‐time quantitative reverse transcription PCR (qRT‐PCR) analysis**

Cells of WT and engineered strains in the late logarithmic phase of cultures were harvested by centrifugation at 5000 ×g for 5 min at 4 ℃ and then flash frozen by liquid nitrogen. Real‐time quantitative reverse transcription PCR (qRT‐PCR) analysis were provided by Yuelixuan Technology Co., Ltd (Beijing, China). The *gyrB* gene encoding DNA gyrase was used as the reference gene due to its relatively stable expression in all phases of growth ^[5]^. The primers used for qRT-PCR were listed in **Table S8.** The expression levels of the target genes were normalized by reference gene *gyrB* using the 2^−ΔΔCT^ method ^[6]^.

**Cr^6+^ reduction and azo dyes degradation**

The Cr^6+^ reduction was implemented in parallel MFC configuration. Cr^6+^ stock solution was prepared with 0.25M K_2_Cr_2_O_7_ and added in cathodic electrolyte to obtain a final Cr^6+^ concentration of 120 mg l^-1^. Samples were then taken every 12 hours and the Cr^6+^ concentration was determined by the 1,5-diphenylcarbazide (DPC) method^[7]^.

The azo dyes methyl orange and amaranth were filtered and added in the serum vials harboring anodic electrolyte with a final concentration of 100 mg l^-1^. Then, the oxygen was removed by sparging with high purity nitrogen. Afterward, the overnight culture of WT and engineered strains was injected at a final OD_600_ of 0.1. Samples were then taken every 30 minutes.

The microplate reader (Tecan infinite 200 pro) was used for measuring the concentration of methyl orange, amaranth, and Cr^6+^ with absorption wavelength of 475 nm, 522 nm, and 540 nm, respectively.

The first‐order rate constant k (h^-1^) was used to evaluate the reduction rates of different pollutants, which could be calculated by Eq. (7),

$$\ln C_{0}-\ln C_{t}=Kt (7)$$

where C_0_ is the initial concentration (mg l^-1^), C_t_ is the concentration at given interval (mg l^-1^), t is the reaction time (h).

**Supplementary Tables**

**Table S1.** Strains used in this study

| Strain | Description | Source |
| --- | --- | --- |
| *Shewanella oneidensis* |  |  |
| MR-1 | Lake Oneida isolate | Our Lab |
| WT | MR-1 harboring pYYDT | Our Lab |
| AsftsA | MR-1 harboring pYYDT-asftsA | This study |
| AsftsN | MR-1 harboring pYYDT-asftsN | This study |
| AsftsQ | MR-1 harboring pYYDT-asftsQ | This study |
| AsftsZ | MR-1 harboring pYYDT-asftsZ | This study |
| MinC | MR-1 harboring pYYDT-minC | This study |
| MinD | MR-1 harboring pYYDT-minD | This study |
| SlmA | MR-1 harboring pYYDT-slmA | This study |
| SulA | MR-1 harboring pYYDT-sulA | This study |
| LuxR | MR-1 harboring pYYDT-luxR_mut7_ | This study |
| LuxI-slmA | MR-1 harboring pYYDT-luxR_mut7_-slmA | This study |
| LuxI-sulA | MR-1 harboring pYYDT-luxR_mut7_-sulA | This study |
| *E. coli* |  |  |
| WM3064 | *ThrB1004 pro thi rpsL hsdS lacZΔM15RP4-1360Δ(araBAD)567 ΔdapA1341::[erm pir]* | Our Lab |

**Table S2.** Plasmids used in this study

| Plasmid | Description | Source |
| --- | --- | --- |
| pYYDT | *pBBR1, kanR, mob, lacI* | Our Lab |
| pYYDT-asftsA | *pBBR1, kanR, mob, lacI，P_tac_-asftsA* | This Study |
| pYYDT-asftsN | *pBBR1, kanR, mob, lacI，P_tac_-asftsN* | This Study |
| pYYDT-asftsQ | *pBBR1, kanR, mob, lacI，P_tac_-asftsQ* | This Study |
| pYYDT-asftsZ | *pBBR1, kanR, mob, lacI，P_tac_-asftsZ* | This Study |
| pYYDT-minC | *pBBR1, kanR, mob, lacI，P_tac_-minC* | This Study |
| pYYDT-minD | *pBBR1, kanR, mob, lacI，P_tac_-minD* | This Study |
| pYYDT-slmA | *pBBR1, kanR, mob, lacI，P_tac_-slmA* | This Study |
| pYYDT-sulA | *pBBR1, kanR, mob, lacI，P_tac_-sulA* | This Study |
| pYYDT-luxR_mut7_-slmA | *pBBR1, kanR, mob, luxR_mut7_，P_luxI_-BBa_B0064-luxI-BBa_B0064-slmA* | This Study |
| pYYDT-luxR_mut7_-sulA | *pBBR1, kanR, mob, luxR_mut7_，P_luxI_-BBa_B0064-luxI-BBa_B0064-sulA* | This Study |
| pYYDT-luxR-gfp | *pBBR1, kanR, mob, luxR，P_luxI_-BBa_B0034-luxI-gfp* | This Study |
| pYYDT-luxR_4B3_-gfp | *pBBR1, kanR, mob, luxR_4B3_，P_luxI_-BBa_B0034-luxI-gfp* | This Study |
| pYYDT-luxR_mut7_-gfp | *pBBR1, kanR, mob, luxR_mut7_，P_luxI_-BBa_B0034-luxI-gfp* | This Study |
| pYYDT-luxR_mut7_ | *pBBR1, kanR, mob, luxR_mut7_，P_luxI_-BBa_B0034-luxI* | This Study |

**Table S3.** Genes used in this study.

| Gene | Function | Source |
| --- | --- | --- |
| *ftsA* | An actin-like protein associated with the membrane through an amphipathic helix to stabilize the Z-ring. | *S. oneidensis* MR-1 |
| *ftsN* | The last recruit protein during divisome assembly which promotes premature interaction between FtsN and FtsA and back recruits the divisome proteins to the Z ring. | *S. oneidensis* MR-1 |
| *ftsQ* | A bitopic membrane proteins that form a complex, acting as a scaffold for the recruitment of downstream divisome proteins. | *S. oneidensis* MR-1 |
| *ftsZ* | A cell-division determinant and bacterial tubulin homolog, assembled into the Z ring at the center of the cell, provides a scaffold for the assembly of the entire Divisome. | *S. oneidensis* MR-1 |
| *minC* | A septum site-determining protein consists of 231 amino acids, and its N-terminal contains FtsZ binding domain, which directly interacts with FtsZ protein and inhibits the polymerization of FtsZ protein. | *S. oneidensis* MR-1 |
| *minD* | A septum site-determining protein consists of 270 amino acids, which can suppress the cell division and prevent the formation of cell division septum, and bind to ATP complex binds to the membrane. | *S. oneidensis* MR-1 |
| *slmA* | A DNA-activated FtsZ polymerization and nucleoid-associated division inhibitor, binds to both the nucleoid and FtsZ to prevent Z rings from forming over chromosomes prior to their proper duplication and partitioning. | *S. oneidensis* MR-1 |
| *sulA* | A cell division inhibitor, blocking binary division, preventing the assembly of nascent Z rings. | *S. oneidensis* MR-1 |
| *luxR* | Quorum sensing transcription regulator protein response to AHLs | *Vibrio fischerii* |
| *luxR_mut 7_* | A LuxR mutant with lower leakiness | *Vibrio fischerii* |
| *luxR_4B3_* | A LuxR mutant with lower leakiness | *Vibrio fischerii* |
| *luxI* | A signaling molecule acyl-homoserine  lactones (AHLs) synthase | *Vibrio fischerii* |

**Table S4.** asRNA used for translational inhibition in this study.

| Name | Sequence (5’-3’) |
| --- | --- |
| asftsA | CATATGAGGAGGAATTAACCATGCAGTGGTGGTGGTGGTGGTGCCATGGGTTCCTATGTCCAATCCGACGATCAGATTTCTATCTTGGTTTTTCGTCATTAATTATCGGCTCTCTTGTTTGTGCATCGCCCCAGCCTACGGCCAACCCTCTCCAGCACCACCACCACCACCACTGCATGGTTAATTCCTCCTGTCGAC |
| asftsN | CATATGAGGAGGAATTAACCATGCAGTGGTGGTGGTGGTGGTGCCATGGGGTTTTGCGCCCGATTGCGGTCTTCTGTTGGCATAGTCACGATTGCTCATGGCTTACATCCGTTCTAGGGTTTCGATACCTAGTAGATTTAGGCCTTTTTCTCCAGCACCACCACCACCACCACTGCATGGTTAATTCCTCCTGTCGAC |
| asftsQ | CATATGAGGAGGAATTAACCATGCAGTGGTGGTGGTGGTGGTGCCATGGACCTGTGACTTCCTCGCCCGCCAGTGTCGCCTCTTATCACTCCAAGACACGTTTGTTTCCACCTTTCAACGACACTACTAAAAACCACAGAAAATCGGCCCTCCAGCACCACCACCACCACCACTGCATGGTTAATTCCTCCTGTCGAC |
| asftsZ | CATATGAGGAGGAATTAACCATGCAGTGGTGGTGGTGGTGGTGCCATGGTACCGCCGCCGCCGCCAACGCCGATGACTTTAATCACCGCGTCGTCTGAGTGAGTGTCCATGATCTCAAACATTGTCTGATCTCCGTGTTGCCTGCGTTACTCCAGCACCACCACCACCACCACTGCATGGTTAATTCCTCCTGTCGAC |

**Table S5.** Primers used for plasmid construction in this study.

| Name | Sequence (5’-3’) |
| --- | --- |
| pYYDT-F | GCCAGGCATCAAATAAAACGAAAGGCT |
| pYYDT-R | CTATGGTCCTTGTTGGTGAAGTGCTCGT |
| SulA-F | TTCACCAACAAGGACCATAGATGAACAAACTATTAGGTAATGCCCC |
| SulA-R | CGTTTTATTTGATGCCTGGCTTAATGAACAGAGCTGAAAAAAGC |
| MinC-F | TTCACCAACAAGGACCATAGATGTCAAAACCTAGCTTAGAGT |
| MinC-R | CGTTTTATTTGATGCCTGGCTTAGAGGGGCAATGATTCA |
| MinD-F | TTCACCAACAAGGACCATAGATGGCACAAATTATTGTTGT |
| MinD-R | CGTTTTATTTGATGCCTGGCTTAGCTACCAAATATCCGT |
| SlmA-F | TTCACCAACAAGGACCATAGATGGCTGTAAGCCCAAAAAT |
| SlmA-R | CGTTTTATTTGATGCCTGGCTTAGCTTTGCAACAGCTGATGCT |
| pYYDT-LuxR-F | CATCTAGTATTTCCCCTCTTTCTCTAGAT |
| PYYDT-LuxR-R | GGATCCGAGGTACCACAGAAATCAT |
| LuxI-sulA-F | TTCTGTGGTACCTCGGATCCTTAATGAACAGAGCTGAAAAAAGC |
| LuxI-sulA-R | AAGAGGGGAAATACTAGATGATGAACAAACTATTAGGTAAT |
| LuxI-slmA-F | TTCTGTGGTACCTCGGATCCTTAGCTTTGCAACAGCTGAT |
| LuxI-slmA-R | AAGAGGGGAAATACTAGATGATGGCTGTAAGCCCAAAAAT |

**Table S6.** Promoters used in this study.

| Name | Sequence (5’-3’) |
| --- | --- |
| P_tac_ | TTGACAATTAATCATCGGCTCGTATAATGTGTGGAATTGTGAGCGGATAACAATTTCACACAGGAAACAGCCAGTCCGTTTAGGTGTTTTCACGAGCACTTCACCAACAAGGACCATAG |
| P_LuxI_- P_LuxR_ | GGTACCTTTCCCCTCTTTAATGAATTCATTCGACTATAACAAACCATTTTCTTGCGTAAACCTGTACGATCCTACAGGTGCTTATGTTAAGTAATTGTATTCCCAGCGATACAATAGTGTGACAAAAATCCAATTTATTAGAATCAAATGTCAATCCATTACCGTTTTAATGATCTACAACACTCAAAACTTACGTCAAACTATCGGTAAAGATAAAGAAATGGGT |

**Table S7.** Sequence of different ribosome binding sites (RBS) used in this study.

| Identifier in iGEM | Sequence | Measured strength in *E. coli* |
| --- | --- | --- |
| BBa_B0029 | GTTTCCTGTGTGAA | 0.764 |
| BBa_B0030 | TTTCTCCTCTTTAAT | 0.6 |
| BBa_B0031 | TCACACAGGAAACC | 0.07 |
| BBa_B0032 | CTTTCCTGTGTGA | 0.3 |
| BBa_B0033 | TCACACAGGAC | 0.01 |
| BBa_B0034 | AAAGAGGAGAAA | 1 |
| BBa_B0035 | ATTAAAGAGGAGAA | 1.124 |
| BBa_B0064 | AAAGAGGGGAAA | 0.35 |

**Table S8.** Primers used for qRT-PCR in this study.

| Name | Sequence (5’-3’) |
| --- | --- |
| gryB-F | CATGGGTGAGAAACTAAACGACT |
| gryB-R | GCAGCATCGACAATCTTACCA |
| ftsA-F | GGAACCTCTAAAGTCGCAGTGA |
| ftsA-R | TACCAAGGCCGACTATGCTG |
| ftsN-F | GAGGTGAAGCCAAAAGAACCTGC |
| ftsN-R | CTTTCTTCGGCGCTTCAACAACA |
| ftsQ-F | ACAGGATTTAATGCAACGGAG |
| ftsQ-R | TGATGCGCGATACACCCAA |
| ftsZ-F | GAATTCGAAACTGTCGGTAACCA |
| ftsZ-R | TACACGTAGTTCGTCGCTCA |
| minC-F | CGGGTAAACAAACTGCGTCT |
| minC-R | GCCATTACCTACCGCACCAA |
| minD-F | ATTATTACCACCAACCCGGAAG |
| minD-R | TGATTTCGACGCCAGAATGC |
| slmA-F | AGATACGATGAAACGCTGTC |
| slmA-R | AATAACGTACTAATGCGGCTAC |
| sulA-F | CCAATTGTCACTATGCCAACCC |
| sulA-R | CCTGCGTTTGCCAATAGCTG |
| luxR-F | CACGCTTGTATGAACATCC |
| luxR-R | TTTACCTTCACAAGCCCAA |
| luxR_mut7_-F | GTTATCTTTCGCTCACTCT |
| luxR_mut7_-R | CAGATTTGTTGTTAGCGAT |
| luxI-F | TCTGCTCCAAAAGATCCAA |
| luxI-R | GCGTGTTTGTAGATAGCTTC |

**Table S9.** The summary and description of differential expressing genes detected in the transcriptomics study. FPKM: Fragments Per Kilobase of transcript per Million mapped reads, which was calculated using software RSEM. Fold change, fold differences in gene transcriptional levels (SulA vs. WT), log_2_(Fold Change) = log_2_[FPKM (SulA)]- log_2_[FPKM (WT)].

| Function | Gene ID | Symbol | Description | FPKM(SulA) | FPKM(WT) | log2(Fold Change) |
| --- | --- | --- | --- | --- | --- | --- |
| Lactate utilization | HRJ35_RS09660 | *dld* | NADH-independent D-lactate dehydrogenase | 638.85 | 887.6333333 | -0.47449 |
|  | HRJ35_RS07135 | *ldhA* | NADH-dependent D-lactate dehydrogenase | 171.2933333 | 253.8833333 | -0.5677 |
|  | HRJ35_RS09665 | *lldP* | L-lactate permease | 108.36 | 430.1533333 | -1.98902 |
|  | HRJ35_RS09645 | *lldG* | L-lactate dehydrogenase complex protein LldG | 179.0666667 | 295.67 | -0.72349 |
|  | HRJ35_RS09650 | *lldF* | L-lactate dehydrogenase iron-sulfur cluster-binding protein LldF | 349.5666667 | 650.36 | -0.89567 |
|  | HRJ35_RS09655 | *lldE* | L-lactate dehydrogenase complex protein LldE | 435.2233333 | 685.6033333 | -0.65562 |
|  | HRJ35_RS18800 | *lldR* | Transcriptional regulator of L-lactate utilization LldR | 43.35 | 44.14333333 | -0.02616 |
| ATP synthesis | HRJ35_RS02545 | *atpG* | F0F1 ATP synthase subunit gamma | 2782.586667 | 4180.82 | -0.58736 |
|  | HRJ35_RS02550 | *atpA* | F0F1 ATP synthase subunit alpha | 2205.176667 | 3352.89 | -0.60451 |
|  | HRJ35_RS02555 | *atpH* | F0F1 ATP synthase subunit delta | 1302.396667 | 2413.816667 | -0.89015 |
|  | HRJ35_RS02560 | *atpF* | F0F1 ATP synthase subunit B | 853.5966667 | 1717.983333 | -1.00909 |
|  | HRJ35_RS02565 | *atpE* | F-ATPase subunit c | 580.5033333 | 1027.09 | -0.82319 |
|  | HRJ35_RS02570 | *atpB* | F0F1 ATP synthase subunit A | 1912.536667 | 3515.346667 | -0.87818 |
|  | HRJ35_RS02575 | *atpI* | ATP synthase assembly protein AtpI | 428.6066667 | 791.4233333 | -0.8848 |
| NADH dehydrogenase | HRJ35_RS06810 | *nqrA* | Na^+^-translocating NADH-quinone reductase subunit A | 727.16 | 454.5266667 | 0.677908 |
|  | HRJ35_RS06815 | *nqrB* | NADH:ubiquinone reductase (Na(+)-transporting) subunit B | 802.0533333 | 340.7233333 | 1.235097 |
|  | HRJ35_RS06820 | *nqrC* | Na^+^-translocating NADH-quinone reductase subunit C | 723.9733333 | 260.2133333 | 1.476242 |
|  | HRJ35_RS06825 | *nqrD* | Na^+^-translocating NADH-quinone reductase subunit D NqrD | 362.7233333 | 126.1666667 | 1.523539 |
|  | HRJ35_RS06830 | *nqrE* | Na^+^-translocating NADH-quinone reductase subunit E NqrE | 457.1866667 | 132.36 | 1.788316 |
|  | HRJ35_RS06835 | *nqrF* | NADH:ubiquinone reductase (Na(+)-transporting) subunit F | 340.4433333 | 107.62 | 1.661468 |
| Menaquinone biosynthetic pathway | HRJ35_RS22075 | *ubiE* | Bifunctional demethylmenaquinone methyltransferase/2-methoxy-6-polyprenyl-1,4-benzoquinol methylase UbiE | 658.66 | 347.4066667 | 0.922908 |
|  | HRJ35_RS02370 | *menF* | Isochorismate synthase | 482.1366667 | 313.8833333 | 0.619214 |
|  | HRJ35_RS01710 | *menD* | 2-succinyl-5-enolpyruvyl-6-hydroxy-3-cyclohexene-1-carboxylic-acid synthase I | 245.1166667 | 175.0866667 | 0.485399 |
|  | HRJ35_RS01720 | *menC* | O-succinylbenzoate synthase | 98.96666667 | 61.25 | 0.692233 |
|  | HRJ35_RS01725 | *menE* | O-succinylbenzoate-CoA ligase | 50.31666667 | 26.77333333 | 0.91024 |
|  | HRJ35_RS11420 | *menA* | 1,4-dihydroxy-2-naphthoate polyprenyltransferase | 143.8733333 | 48.06 | 1.581891 |
| *c*-Cytochromes | HRJ35_RS10740 | *mtrA* | Outer-membrane decaheme c-type cytochrome | 4846.85 | 2171.466667 | 1.158378 |
|  | HRJ35_RS10735 | *mtrB* | MtrB/PioB family decaheme-associated outer membrane protein | 4824.426667 | 2292.82 | 1.073234 |
|  | HRJ35_RS10745 | *mtrC* | Outer-membrane decaheme *c*-type cytochrome | 4428.6 | 2246.88 | 0.978928 |
|  | HRJ35_RS10750 | *omcA* | OmcA/MtrC family decaheme *c*-type cytochrome | 6398.76 | 4129.97 | 0.631661 |
|  | HRJ35_RS01795 | *cymA* | Inner-membrane tetra-heme cytochrome *c* | 9242.283333 | 4419.526667 | 1.064357 |
| *c*-Cytochrome maturation system | HRJ35_RS03835 | *ccmE* | Cytochrome *c* maturation protein CcmE | 1744.17 | 1228.48 | 0.505666 |
|  | HRJ35_RS03840 | *ccmD* | Heme exporter protein CcmD | 914.75 | 593.42 | 0.624324 |
|  | HRJ35_RS03845 | *ccmC* | Heme ABC transporter permease | 699.18 | 425.9766667 | 0.71489 |
|  | HRJ35_RS03850 | *ccmB* | Heme exporter protein CcmB | 436.2166667 | 274.64 | 0.667503 |
|  | HRJ35_RS03855 | *ccmA* | Cytochrome *c* biogenesis heme-transporting ATPase CcmA | 1396.436667 | 1108.903333 | 0.332617 |
| Heme biosynthetic pathway | HRJ35_RS00475 | *hemC* | Hydroxymethylbilane synthase | 1380.383333 | 565.0966667 | 1.288499 |
|  | HRJ35_RS00480 | *hemD* | Uroporphyrinogen-III synthase | 599.8033333 | 216.5933333 | 1.469501 |
|  | HRJ35_RS00485 | *hemX* | Uroporphyrinogen III methylase | 189.8166667 | 120.04 | 0.661091 |
|  | HRJ35_RS01470 | *hemW* | Coproporphyrinogen III oxidase family protein | 4166.85 | 1924.013333 | 1.114838 |
|  | HRJ35_RS02455 | *hemN* | Oxygen-independent coproporphyrinogen III oxidase | 3105.36 | 1070.98 | 1.535829 |
|  | HRJ35_RS04685 | *hemE* | Uroporphyrinogen decarboxylase | 1575.246667 | 451.3066667 | 1.803398 |
|  | HRJ35_RS11910 | *hemH* | Ferrochelatase | 326.4833333 | 177.2666667 | 0.881088 |
| Riboflavin biosynthetic pathway | HRJ35_RS15710 | *ribA* | GTP cyclohydrolase II | 313.3783 | 279.3834333 | 0.165659 |
|  | HRJ35_RS03270 | *ribB* | Bifunctional 3,4-dihydroxy-2-butanone-4-phosphate synthase/GTP cyclohydrolase II | 330.6920867 | 372.0048667 | -0.16983 |
|  | HRJ35_RS18845 | *ribD* | Bifunctional diaminohydroxyphosphoribosylaminopyrimidine deaminase/5-amino-6-(5-phosphoribosylamino) uracil reductase | 192.8819667 | 163.4237333 | 0.239101 |
|  | HRJ35_RS18830 | *ribH* | RecName: Full=6,7-dimethyl-8-ribityllumazine synthase; Short=DMRL synthase; Short=LS; Short=Lumazine synthase | 646.4470667 | 596.2225667 | 0.116681 |
|  | HRJ35_RS19145 | *ribF* | Bifunctional riboflavin kinase/FAD synthetase | 363.0698 | 335.5141333 | 0.113874 |
|  | HRJ35_RS13125 | *ribC* | Riboflavin synthase | 79.39745667 | 94.52650333 | -0.25163 |
|  | HRJ35_RS18840 | *ribE* | Riboflavin synthase | 183.7925667 | 172.0500667 | 0.09525 |
| Outer-membrane porin proteins | HRJ35_RS01985 | *ompR* | two-component system response regulator OmpR | 177.59 | 193.27 | -0.12207 |
|  | HRJ35_RS16815 | *ompS* | porin protein | 12.95333333 | 14.32 | -0.14471 |
|  | HRJ35_RS00510 | *tolC* | TolC family outer membrane protein | 131.8133333 | 207 | -0.65113 |
|  | HRJ35_RS10160 | *ompH* | outer membrane protein OmpH | 284.46 | 362.9333333 | -0.35148 |
|  | HRJ35_RS10155 | *bamA* | outer membrane protein assembly factor BamA | 361.4566667 | 569.3233333 | -0.65543 |
|  | HRJ35_RS02285 | *torF* | TMAO reductase system outer membrane porin TorF | 8.34 | 10.56666667 | -0.3414 |
|  | HRJ35_RS04090 | *phoE* | porin protein | 18.33333333 | 21.15 | -0.20619 |
|  | HRJ35_RS10315 | *ompW* | outer membrane protein OmpW | 4897.103333 | 8666.623333 | -0.82354 |
|  | HRJ35_RS00515 | *oprF* | OmpA family protein | 138.3066667 | 237.9966667 | -0.78307 |
| Biofilm formation | HRJ35_RS07675 | *bolA* | BolA family transcriptional regulator^[8]^ | 188.3366667 | 108.8666667 | 0.790752 |
|  | HRJ35_RS10200 | *dgcS* | GGDEF domain-containing diguanylate cyclase^[9]^ | 46.13 | 33.82666667 | 0.447544 |
|  | HRJ35_RS17405 | *capD* | polysaccharide biosynthesis protein | 179.6233333 | 305.6433333 | -0.76687 |
|  | HRJ35_RS21985 | *mxdD* | Periplasmic protein of expolysaccharide biosynthesis cluster mxdD^[10]^ | 55.20333333 | 83.09 | -0.58992 |
|  | HRJ35_RS06375 | *metXA* | CBS domain-containing protein | 150.5066667 | 305.9433333 | -1.02344 |
|  | HRJ35_RS17435 | *SO_3177* | Formyl transferase^[11]^ | 160.73 | 242.5966667 | -0.59392 |
|  | HRJ35_RS18035 | *SO_3303* | Cell surface protein | 10.17333333 | 5.093333333 | 0.99811 |
|  | HRJ35_RS04610 | *pilA* | Type IV pilin protein PilA | 8951.356667 | 4122.413333 | 1.118617 |
|  | HRJ35_RS00350 | *motB* | Stator-force generator of H^+^ coupled flagellar motor MotB | 156.88 | 76.25 | 1.040852 |
|  | HRJ35_RS17770 | *flgF* | Flagellar basal-body rod protein FlgF | 188.8866667 | 80.23666667 | 1.235187 |
| Global regulation | HRJ35_RS05555 | *crp* | cAMP-responsive regulator of catabolite repression Crp^[12]^ | 6755.713333 | 1693.956667 | 1.995711 |
|  | HRJ35_RS21195 | *arcA* | Two component signal transduction system controlling aerobic respiration response regulator^[13]^ | 2862.993333 | 2315.13 | 0.306431 |
|  | HRJ35_RS08740 | *hptA* | Histidine-containing phosphotransfer domain protein HptA of respiration control (Arc) regulatory system^[13]^ | 673.5766667 | 306.9 | 1.134074 |
|  | HRJ35_RS05325 | *arcS* | Sensor kinase of respiration control (Arc) regulatory system^[13]^ | 41.3 | 60.18666667 | -0.5433 |
| Ribonucleoside-diphosphate reductases | HRJ35_RS13675 | *nrdA* | Ribonucleoside-diphosphate reductase subunit alpha | 648.2366667 | 617.1766667 | 0.070837 |
|  | HRJ35_RS13680 | *nrdB* | Ribonucleotide-diphosphate reductase subunit beta | 186.4433333 | 176.6766667 | 0.077626 |
|  | HRJ35_RS18850 | *nrdR* | Transcriptional repressor NrdR | 117.9766667 | 140.3466667 | -0.25049 |
|  | HRJ35_RS15720 | *nrdG* | Anaerobic ribonucleoside-triphosphate reductase activating protein NrdG | 301.1433333 | 212.54 | 0.502716 |
|  | HRJ35_RS15725 | *nrdD* | Anaerobic ribonucleoside-triphosphate reductase | 941.6566667 | 515.77 | 0.868473 |
| Cell division proteins | HRJ35_RS00025 | *ftsZ* | Cell division protein FtsZ | 913.8133333 | 1055.803333 | -0.20837 |
|  | HRJ35_RS00030 | *ftsA* | Cell division protein FtsA | 273.52 | 254.22 | 0.105569 |
|  | HRJ35_RS00035 | *ftsQ* | FtsQ-type POTRA domain-containing protein | 118.4066667 | 132.8166667 | -0.16569 |
|  | HRJ35_RS00050 | *ftsW* | Cell division protein FtsW | 137.14 | 125.9233333 | 0.123104 |
|  | HRJ35_RS00075 | *ftsI* | Peptidoglycan glycosyltransferase FtsI | 140.2133333 | 191.5333333 | -0.44997 |
|  | HRJ35_RS00080 | *ftsL* | Cell division protein FtsL | 116.9766667 | 91.09333333 | 0.360803 |
|  | HRJ35_RS01760 | *ftsX* | Cell division protein FtsX | 32.36 | 61.69333333 | -0.9309 |
|  | HRJ35_RS01765 | *ftsE* | Cell division ATP-binding protein FtsE | 61.62333333 | 79.22 | -0.36239 |
|  | HRJ35_RS01770 | *ftsY* | Signal recognition particle-docking protein FtsY | 33.71333333 | 34.94 | -0.05156 |
|  | HRJ35_RS08155 | *ftsH* | ATP-dependent zinc metalloprotease FtsH | 1095.356667 | 1117.476667 | -0.02884 |
|  | HRJ35_RS13175 | *ftsK* | DNA translocase FtsK | 95.04666667 | 98.42333333 | -0.05036 |
|  | HRJ35_RS18700 | *ftsB* | Cell division protein FtsB | 90.51333333 | 56.69 | 0.675036 |
|  | HRJ35_RS04195 | *zapB* | Cell division protein ZapB | 793.6966667 | 326.8366667 | 1.280018 |
|  | HRJ35_RS04585 | *zapD* | Cell division protein ZapD | 45.13333333 | 43.94666667 | 0.03844 |
|  | HRJ35_RS06245 | *zapA* | Cell division protein ZapA | 237.8533333 | 159.54 | 0.576154 |
|  | HRJ35_RS14535 | *zapC* | Cell division protein ZapC | 405.3433333 | 370.0366667 | 0.131476 |
|  | HRJ35_RS16030 | *zipA* | Cell division protein ZipA | 219.4366667 | 194.9766667 | 0.170503 |
|  | HRJ35_RS20985 | *zapE* | Cell division protein ZapE | 53.05 | 35.26 | 0.58932 |
| Cell division inhibitors | HRJ35_RS01855 | *sulA* | Cytoplasmic division inhibitor | 1922.516667 | 155.91 | 3.624211 |
|  | HRJ35_RS00200 | *slmA* | Nucleoid occlusion factor SlmA | 162.9966667 | 75.48333333 | 1.110612 |
|  | HRJ35_RS14460 | *minC* | Septum site-determining protein MinC | 52.27666667 | 39.08333333 | 0.419614 |
|  | HRJ35_RS14465 | *minD* | Septum site-determining protein MinD | 294.9133333 | 217.1 | 0.441931 |
|  | HRJ35_RS14470 | *minE* | Cell division topological specificity factor | 364.5833333 | 355.2033333 | 0.037603 |
| Cell cytoskeletal proteins | HRJ35_RS21640 | *mreB* | Cell wall structural complex component MreB | 284.8266667 | 202.9766667 | 0.48877 |
|  | HRJ35_RS21635 | *mreC* | Rod shape-determining protein MreC | 55.57666667 | 52.22333333 | 0.089785 |
|  | HRJ35_RS21630 | *mreD* | Rod shape-determining protein MreD | 37.31 | 47.42 | -0.34593 |
|  | HRJ35_RS07975 | *mrdB* | Rod shape-determining protein RodA | 25.36 | 55.91666667 | -1.14072 |
|  | HRJ35_RS18075 | *rodZ* | Rod shape-determining protein RodZ | 229.9066667 | 304.3433333 | -0.40465 |

**Supplementary Figures**


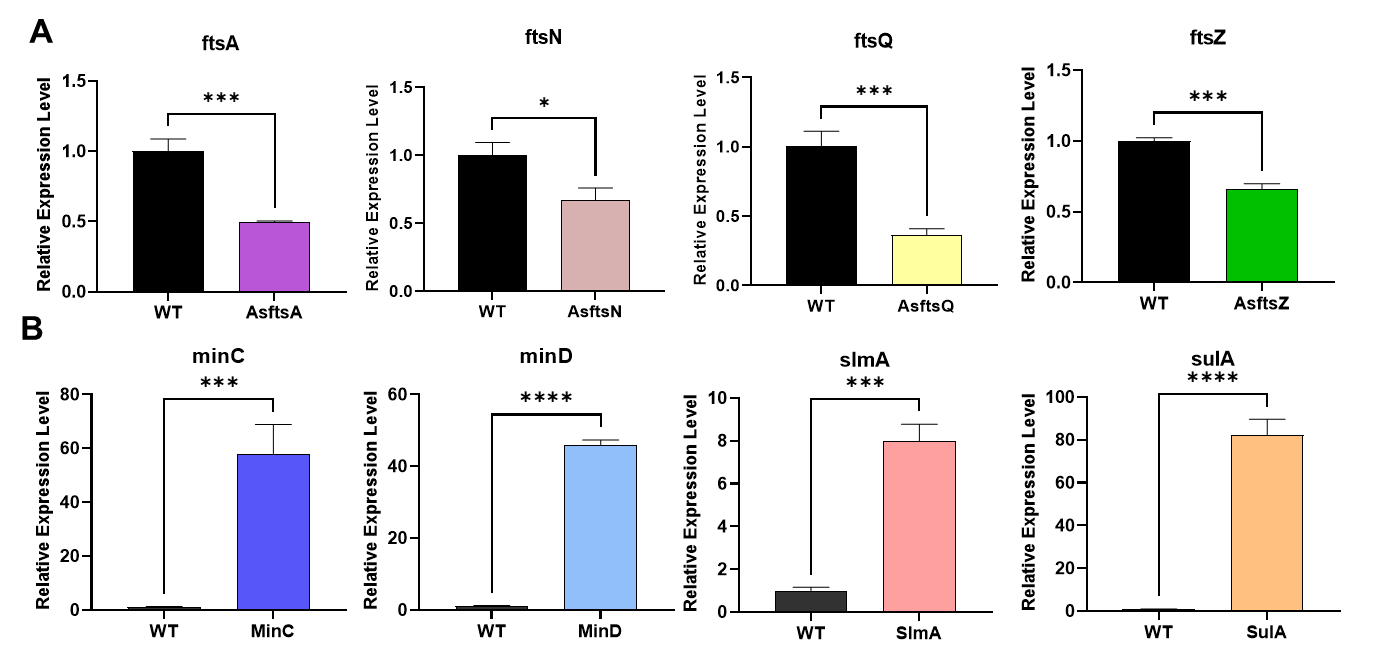


**Figure S1**. Relative transcriptional levels of target genes in the four engineered strains expressing asRNAs (**A**) and division inhibitors (**B**), which were normalized as the expression levels that in the WT. The data were shown as the mean ± SD from three independent replicates. Significant difference was analyzed by ordinary one-way ANOVA method.

**
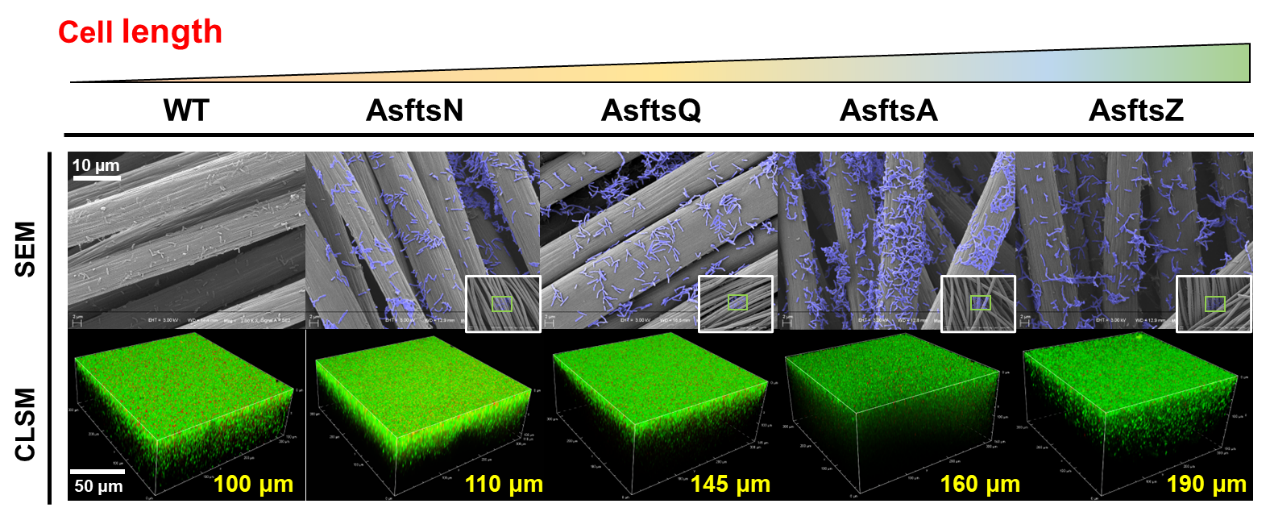
**

**Figure S2**. SEM and CLSM images of the WT and four elongated *S. oneidensis* strains overexpressing division inhibitors, ordered from left to right by increasing cell length (scale bar: 10 µm for SEM; 50 µm for CLSM images).


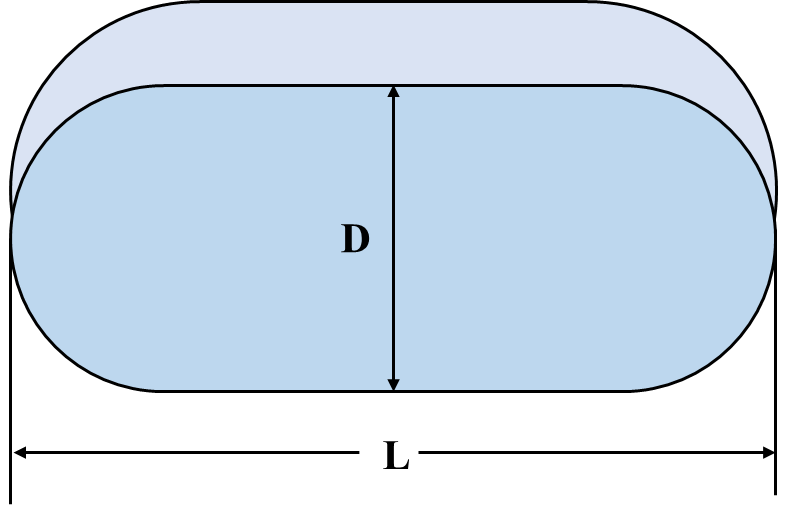


**Figure S3**. The model of rod-shape for calculation of dimensional parameters. L is the cell length from pole to pole. D is the cell width from cell midline to cell midline.


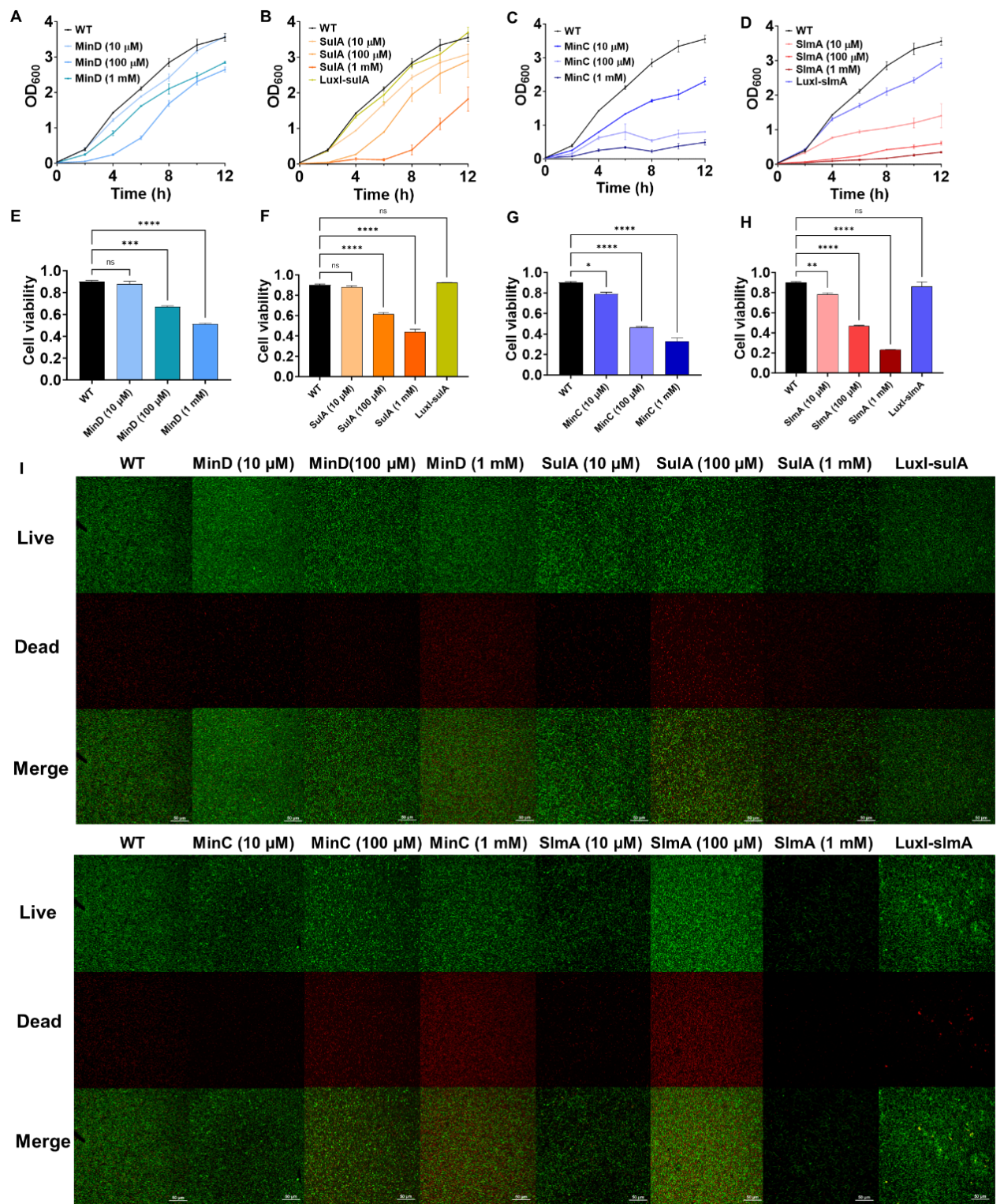


**Figure S4. Cell growth and viability of the WT and the engineered strains overexpressing division inhibitors under the induction of different concentrations of IPTG.** (A) Growth curves of WT and the engineered strain MinD under the induction of 10 µM, 100 µM, and 1 mM IPTG. (B) Growth curves of WT, the engineered strain SulA under the induction of 10 µM, 100 µM, and 1 mM IPTG, and the QS-regulated strain LuxI-sulA. (C) Growth curves of WT and the engineered strain MinC under the induction of 10 µM, 100 µM, and 1 mM IPTG. (D) Growth curves of WT, the engineered strains SlmA under the induction of 10 µM, 100 µM, and 1 mM IPTG, and the QS-regulated strain LuxI-slmA. (E) Cell viabilities of strains WT and MinD under the induction of 10 µM, 100 µM, and 1 mM IPTG. (F) Cell viabilities of strains WT, SulA under the induction of 10 µM, 100 µM, and 1 mM IPTG, and LuxI-sulA (G) Cell viabilities of strains WT and MinC under the induction of 10 µM, 100 µM, and 1 mM IPTG. (H) Cell viabilities of strains WT, SlmA under the induction of 10 µM, 100 µM, and 1 mM IPTG, and LuxI-slmA. Data were presented by three independent biological replicates as means ± SD. Significant difference was analyzed by the ordinary one-way ANOVA method. (I) CLSM observation of the live/dead staining of WT, the engineered strains MinC, MinD, SulA, and SlmA, under the induction of different concentration of IPTG, as well as the QS-based strains LuxI-sulA and LuxI-slmA (scale bar: 50 µm). Green fluorescence represented live cells labeled with SYTO 9, red fluorescence represented dead cells labeled with propidium iodide (PI), and merge represents all of the live and dead cells. The excitation/emission wavelengths were 480/500 nm for SYTO 9 stain and 490/635 nm for PI.

**
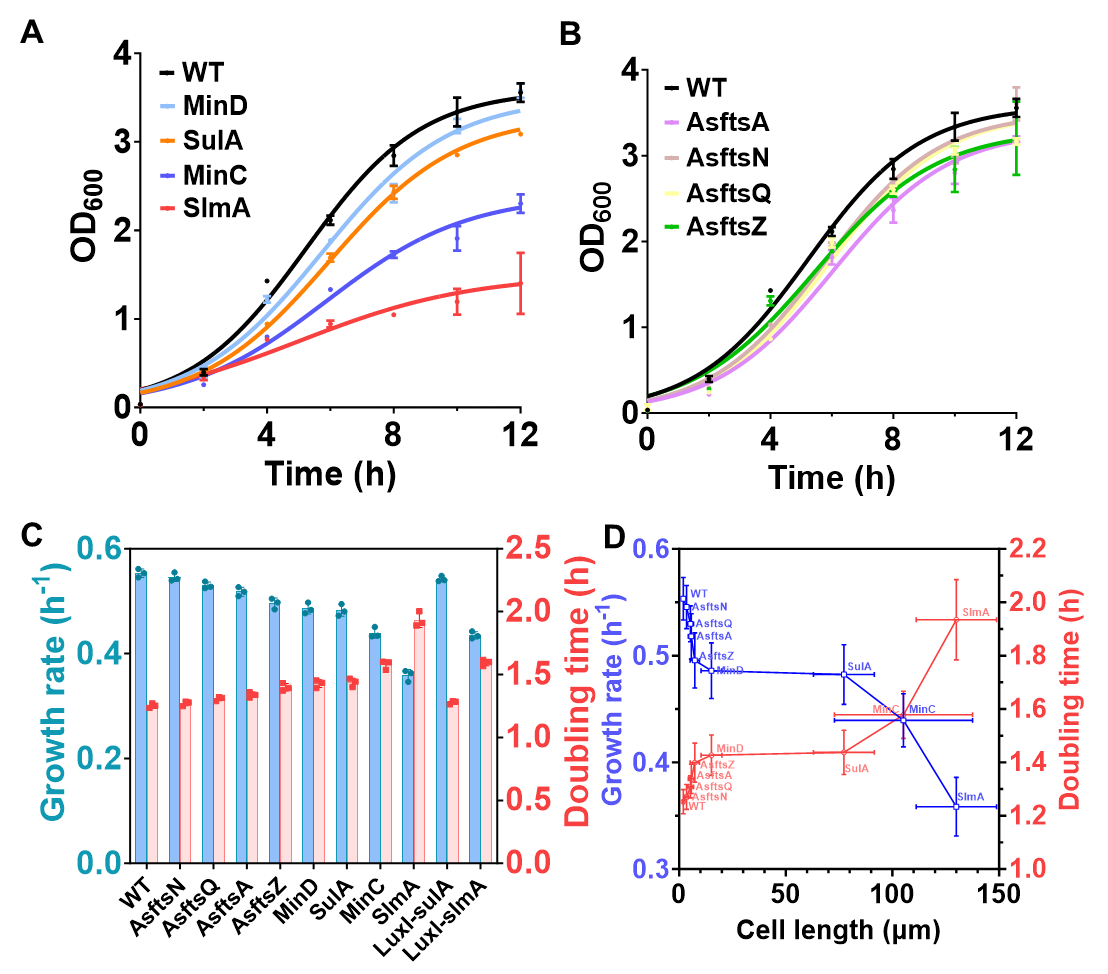
**

**Figure S5**. **Quantification of the correlation between cell length and cell growth.** (A) Growth curves of WT and the elongated strains MinD, SulA, MinC, and SlmA cultured in LB medium, under the induction of 10 µM IPTG, respectively, which were fitted with the Logistic curves by using the GraphPad Prism 10.1.2 software. (B) Growth curves of the engineered strains AsftsA, AsftsN, AsftsQ, and AsftsZ under induction of 1 mM IPTG, which were fitted with the Logistic curves by using the GraphPad Prism 10.1.2 software. (C) Calculation of the specific growth rate and doubling time of the wild-type (WT) and the eight elongated *S. oneidensis* strains expressing the division inhibitors. (**D**) Specific growth rates and doubling time corresponding to different cell length. Data were presented by three independent biological replicates as means ± SD.


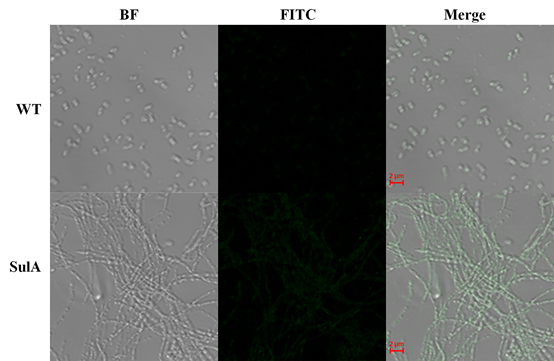


**Figure S6**. Immunofluorescence localization of cell-surface *c*-type cytochrome MtrC of WT and elongated strain SulA.


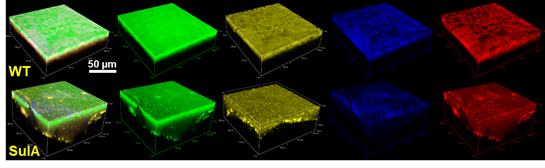


**Figure S7.** Comparison of extracellular polymeric substances (EPS) thickness and components of WT and the filamentous strain SulA-based biofilm. Extracellular protein, α-polysaccharide, β-polysaccharide, and extracellular DNA were stained by fluorescein isothiocyanate (FITC), Concanavalin (ConA), Calcofluor white (CW), and propidium iodide (PI), and shown in green, yellow, blue, and red, respectively (scale bar: 50 µm).

**Figure S8**. UV/vis spectral characterization of the *c*-Cyts levels from the EPS of the WT and elongated *S. oneidensis* strain SulA.


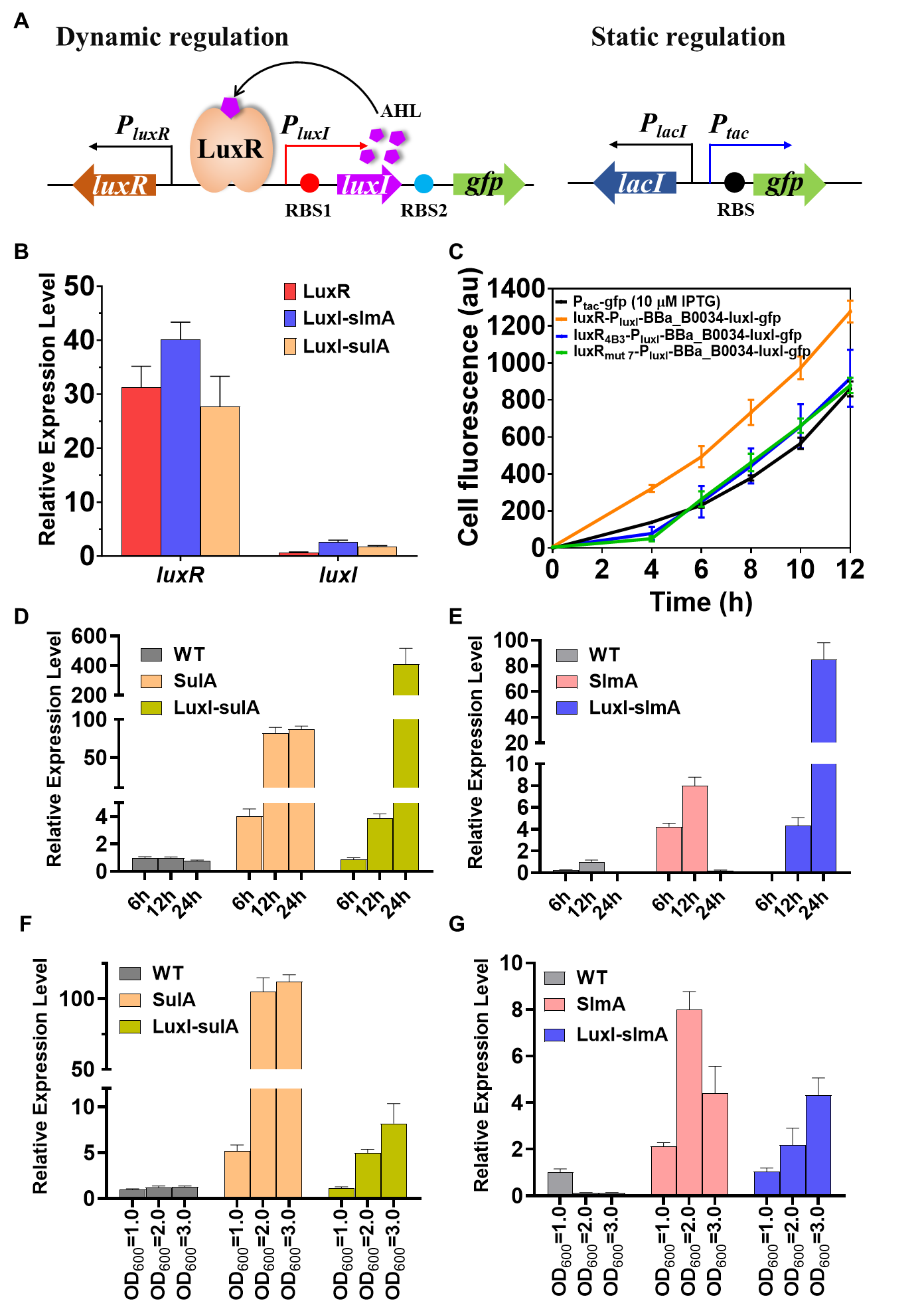


**Figure S9. Design and verification of quorum sensing (QS)-based dynamic regulation system by fluorescence detection and qPCR analyses.** (A) Design of a quorum sensing (QS)-based positive-feedback-loop genetic circuit for dynamic regulation by placing both *luxI* and *gfp* genes under the control of the synthetic RBS (RBS1 and RBS2, for the genes *luxI* and *gfp*, respectively) and the LuxR-regulated promoter P_lux_. P_tac_-*gfp* was the static regulation circuit induced by 10 µM IPTG. (B) Relative transcriptional levels of the target genes *luxR* and *luxI* in engineered strains harboring the QS system cultured in LB medium, which were normalized as the expression levels of *gyrB*. (C) Verification of the QS -based genetic circuits by cell fluorescence intensity detection of strains harboring P_tac_-gfp, LuxR-luxI-gfp, LuxR_4B3_-luxI-gfp, and LuxR_mut7_-luxI-gfp cultured in LB medium. (D) Relative transcriptional levels of the gene *sulA* in the engineered strain SulA and the QS-based strain LuxI-sulA at different growth phases (6h, 12h, and 24h) cultured in LB medium, which were normalized as the expression levels that in WT at 12 h. (E) Relative transcriptional levels of *slmA* in the engineered strain SlmA and the QS-based strain LuxI-slmA at different growth phases (6h, 12h, and 24h) cultured in LB medium, which were normalized as the expression levels that in WT at 12 h. (F) Relative transcriptional levels of the gene *sulA* in the engineered strain SulA and the QS-based strain LuxI-sulA at different cell densities (OD_600_=1, 2, and 3) cultured in LB medium, which were normalized as the expression levels that in WT at OD_600_=1. (G) Relative transcriptional levels of *slmA* in the engineered strain SlmA and the QS-based strain LuxI-slmA at different cell densities (OD_600_=1, 2, and 3) cultured in LB medium, which were normalized as the expression levels that in WT at OD_600_=1. Data were presented by three independent biological replicates as means ± SD.

**
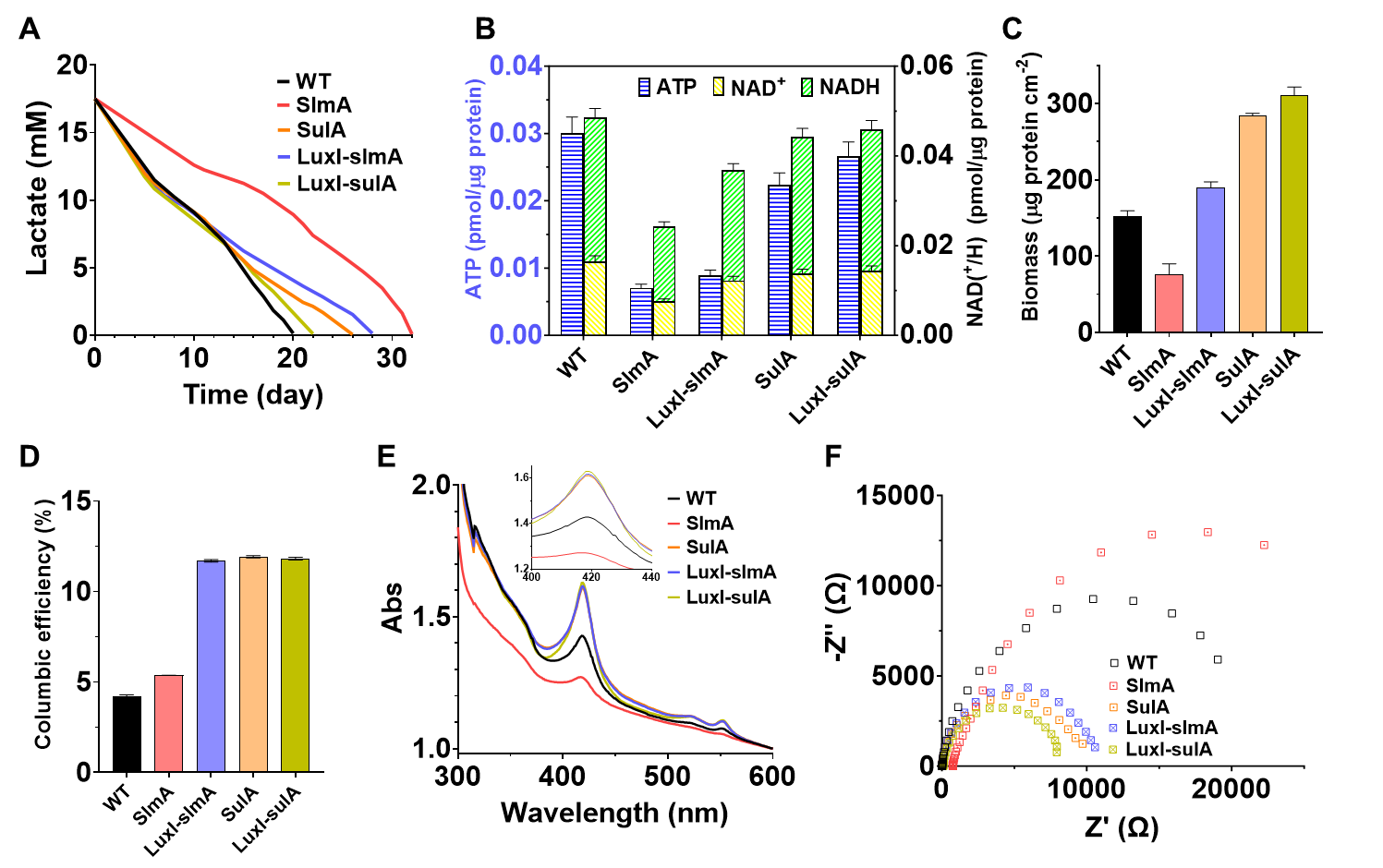
**

**Figure S10. Electrophysiological performance of QS-regulated elongated strains.** (A) Lactate consumption profile of the strains WT, SulA, SlmA, and the QS-regulated strains in MFC. (B) ATP assay and NAD(^+^/H) measurement of the strains WT, SlmA, LuxI-slmA, SulA, and LuxI-sulA. (C) Electrode-attached biomass contents of the strains WT, SlmA, LuxI-slmA, SulA, and LuxI-sulA. (D) Columbic efficiencies of the strains WT, SlmA, LuxI-slmA, SulA, and LuxI-sulA. (E) UV/vis spectral characterization of the *c*-Cyts level of strains WT, SulA, SlmA, and QS-based strains. Insert was the magnification of the section of 400-420 nm. (F) Nyquist plots of electrochemical impedance spectroscopy (EIS) of WT, SulA, SlmA, and QS-based strains. Z' and Z" refer to the real part and imaginary part of the impedance. Data were presented by three independent biological replicates as means ± SD.


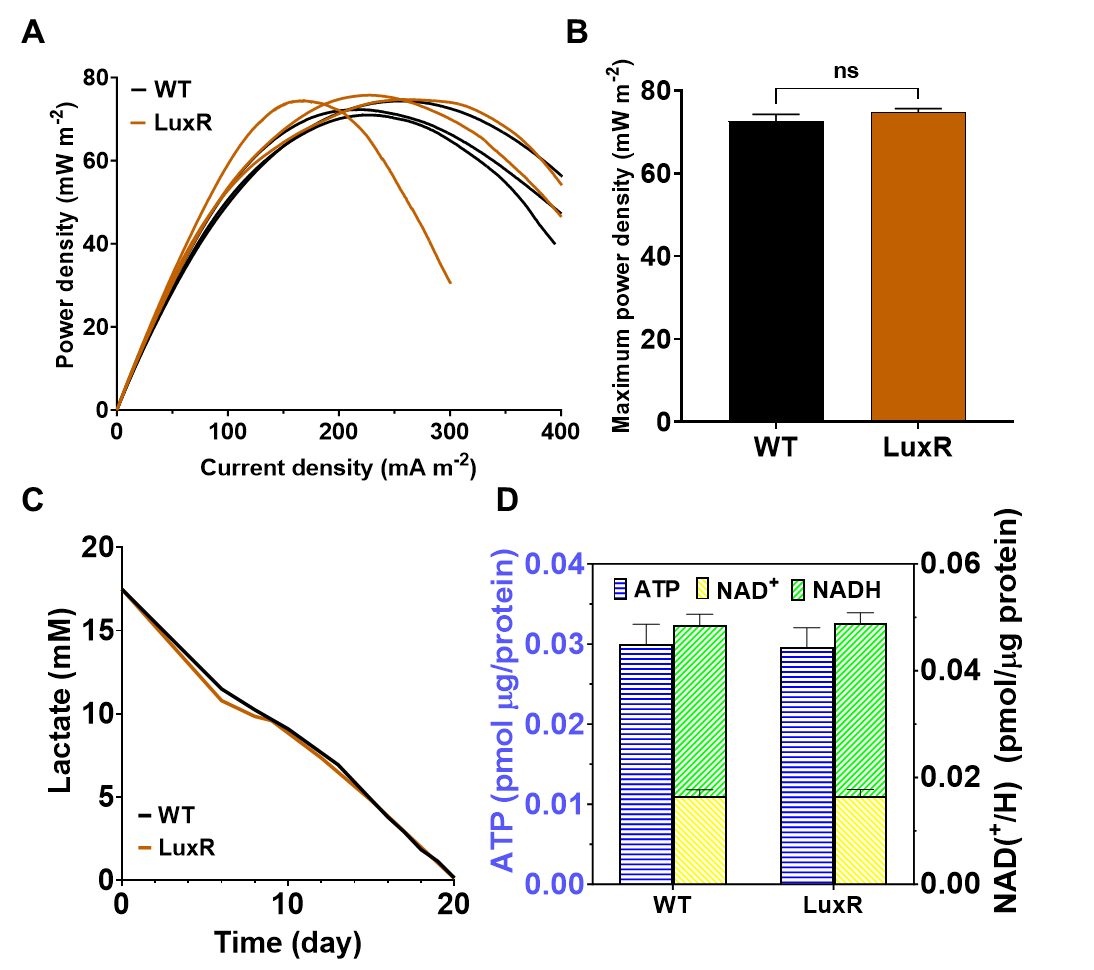


**Figure S11**. **Elimination of quorum sensing’s effect on EET of the normal-size strain.** (A) Power density curves of the strains WT and LuxR that expressed the quorum sensing (QS) system. Three biological replicates were examined for each strain. (B) Comparison of maximum power densities of the strains WT and LuxR. Significant difference was determined by two-tailed t test. (C) Lactate consumption curves of the strains WT and LuxR. (D) ATP assay and NAD(^+^/H) measurement of the strains WT and LuxR. Data were presented by three independent biological replicates as means ± SD. Significant difference was analyzed by ordinary one-way ANOVA method.


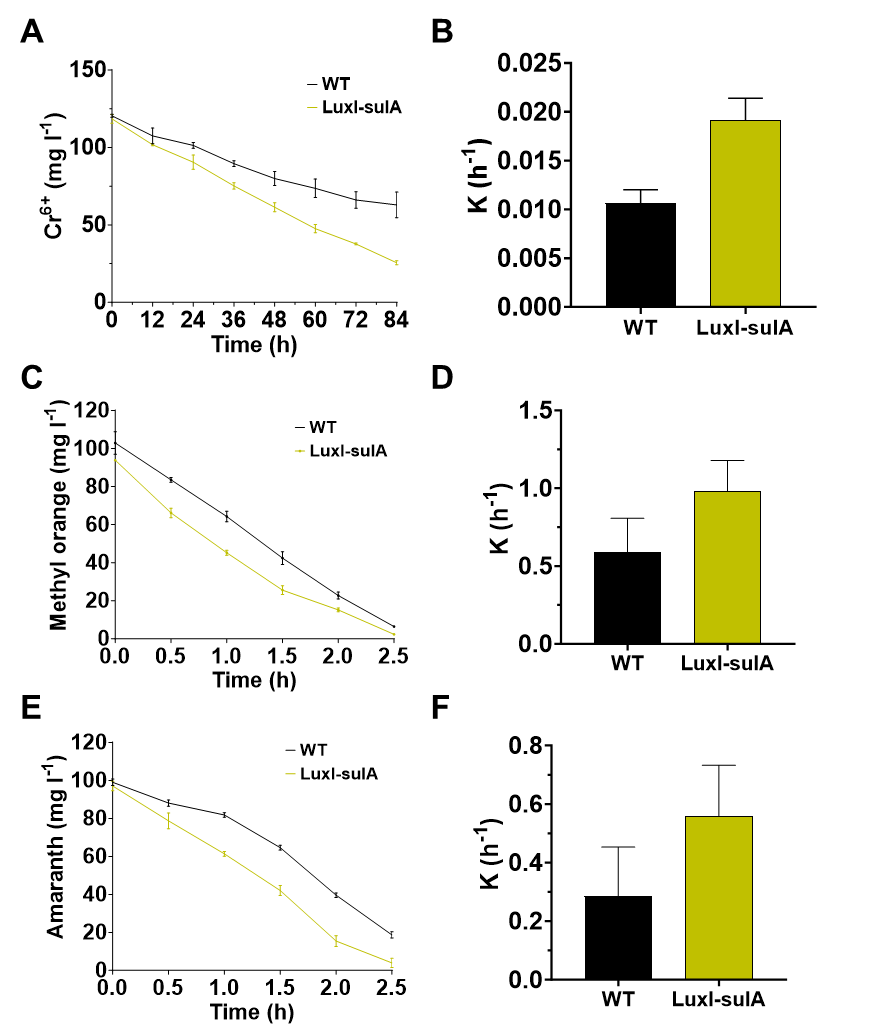


**Figure S12**. Application of the engineered strain for pollution treatment. (A) Cathodic reduction curve of Cr^6+^ of the WT and engineered strain LuxI-sulA. (B) Reduction kinetic constant of Cr^6+^ of WT and LuxI-sulA. (C) Anaerobic degradation curve of methyl orange of WT and LuxI-sulA. (D) Degradation kinetic constant of methyl orange of WT and LuxI-sulA. (E) Anaerobic degradation curve of amaranth of WT and LuxI-sulA. (F) Degradation kinetic constant of amaranth of WT and LuxI-sulA. Data were presented by three independent biological replicates as means ± SD.

**Figure S13.** Normalized power density to electrode-attached biomass of the elongated *S. oneidensis* strains. Data were presented by three independent biological replicates as means ± SD. Significant difference was performed by Ordinary one-way ANOVA method of GraphPad Prism 10.1.2 software.

**Supplementary References**

[1] D. G. Gibson, L. Young, R.-Y. Chuang, J. C. Venter, C. A. Hutchison, H. O. Smith, *Nat. Methods* **2009**, *6*, 343-345.

[2] N. Nakashima, T. Tamura, L. Good, *Nucleic Acids Res.* **2006**, *34*, e138.

[3] J. Guo, G. Yang, Z. Zhuang, Q. Mai, L. Zhuang, *Sci. Total Environ.* **2021**, *797*, 149207.

[4] D. Yan, X. Yang, W. Yuan, *Journal of Power Sources* **2015**, *289*, 26-33.

[5] A. E. Murray, D. Lies, G. Li, K. Nealson, J. Zhou, J. M. Tiedje, *Proc. Natl. Acad. Sci. USA* **2001**, *98*, 9853-9858.

[6] K. J. Livak, T. D. Schmittgen, *Methods* **2001**, *25*, 402-408.

[7] L. F. Oliveira, N. T. Canevari, M. B. B. Guerra, F. M. V. Pereira, C. E. G. R. Schaefer, E. R. Pereira-Filho, *Microchemical Journal* **2013**, *109*, 165-169.

[8] A. V. Silva, M. Edel, J. Gescher, C. M. Paquete, *Front. Microbiol.* **2020**, *11*, 815.

[9] A. Matsumoto, R. Koga, A. Kanaly Robert, A. Kouzuma, K. Watanabe, M. Kivisaar, *Appl. Environ. Microbiol.* **2021**, *87*, e00201-00221.

[10] J. Müller, S. Shukla, K. A. Jost, A. M. Spormann, *BMC Microbiol.* **2013**, *13*, 119.

[11] A. Kouzuma, X. Y. Meng, N. Kimura, K. Hashimoto, K. Watanabe, *Appl. Environ. Microbiol.* **2010**, *76*, 4151-4157.

[12] T. Kasai, A. Kouzuma, K. Watanabe, *Front. Microbiol.* **2017**, *8*, 869.

[13] A. Hirose, A. Kouzuma, K. Watanabe, *Biotechnol. Adv.* **2019**, *37*, 107351.
